# The Exploration of Novel Pharmacophore Characteristics and Multidirectional Elucidation of Structure-Activity Relationship and Mechanism of Sesquiterpene Pyridine Alkaloids from *Tripterygium* Based on Computational Approaches

**DOI:** 10.1155/2021/6676470

**Published:** 2021-03-24

**Authors:** Chengyan Long, Yang Yang, Yong Yang, Sixing Huang, Xiaomei Zhang, Wei Du, Dajian Yang, Yanlei Guo, Li Zhang

**Affiliations:** Chongqing Academy of Chinese Materia Medica, No. 34 Nanshan Road, Nan'an District, Chongqing 400065, China

## Abstract

Sesquiterpene pyridine alkaloids are a large group of highly oxygenated sesquiterpenoids, which are characterized by a macrocyclic dilactone skeleton containing 2-(carboxyalkyl) nicotinic acid and dihydro-*β*-agarofuran sesquiterpenoid, and are believed to be the active and less toxic components of *Tripterygium*. In this study, 55 sesquiterpene pyridine alkaloids from *Tripterygium* were subjected to identification of pharmacophore characteristics and potential targets analysis. Our results revealed that the greatest structural difference of these compounds was in the pyridine ring and the pharmacophore model-5 (Pm-05) was the best model that consisted of three features including hydrogen bond acceptor (HBA), hydrogen bond donor (HBD), and hydrophobic (HY), especially hydrophobic group located in the pyridine ring. It was proposed that 2-(carboxyalkyl) nicotinic acid part possessing a pyridine ring system was not only a pharmacologically active center but also a core of structural diversity of alkaloids from *Tripterygium wilfordii*. Furthermore, sesquiterpene pyridine alkaloids from *Tripterygium* were predicted to target multiple proteins and pathways and possibly played essential roles in the cure of Alzheimer's disease, breast cancer, Chagas disease, and nonalcoholic fatty liver disease (NAFLD). They also had other pharmacological effects, depending on the binding interactions between pyridine rings of these compounds and active cavities of the target genes platelet-activating factor receptor (PTAFR), cannabinoid receptor 1 (CNR1), cannabinoid receptor 1 (CNR2), squalene synthase (FDFT1), and heat shock protein HSP 90-alpha (HSP90AA1). Taken together, the results of this present study indicated that sesquiterpene pyridine alkaloids from *Tripterygium* are promising candidates that exhibit potential for development as medicine sources and need to be promoted.

## 1. Introduction

Traditional Chinese medicine (TCM) and natural products represent a huge source of diverse new drugs due to their potent and highly varied physiological activities. Plants in the genus *Tripterygium*, such as *Tripterygium wilfordii* Hook. f. (TWHF), a typical traditional Chinese medicine plant, have been widely used to treat autoimmune diseases and neurodegenerative diseases including rheumatoid arthritis, systemic lupus erythematosus, and Parkinson's disease in China [1–3]. Chemical and pharmacological studies have demonstrated that alkaloids, diterpenes, triterpenes, and lignans were major bioactive components found in *T. wilfordii* responsible for the overall curative effects [4–6]. Triptolide and celastrol are considered as predominantly active natural products of TWHF and are used as a remedy for inflammatory and autoimmune diseases [7, 8]. As the most promising bioactive compound obtained from TWHF, triptolide has attracted considerable interest recently, especially for its biosynthesis [9]. However, toxicity restricts the further development of triptolide and celastrol [10].

Alkaloids with diverse structures are an important class of chemical components in TWHF [11]. A total of 161 alkaloids have been reported in TWHF, which mainly include sesquiterpene pyridine alkaloids, sesquiterpene nonmacrocyclic lactone alkaloids, and a few other types of alkaloids [12–14]. Importantly, sesquiterpene pyridine alkaloids are the most abundant in TWHF. From the perspective of structural characteristics, they can be divided into two categories: sesquiterpene pyridine alkaloids, in which pyridine ring 2′,3′ position is connected to macrocyclic and pyridine ring 3′,4′ position is connected to macrocyclic. The diversity of the positions of the connectable groups of the sesquiterpene part and the complexity of the substitutable groups lead to a wide variety of compounds.

Sesquiterpene pyridine alkaloids from *Tripterygium* have been extensively studied due to their potential role in a wide spectrum of pharmacological activities, such as anti-inflammatory, antimicrobial and antitumor activities. TWHF alkaloids are less toxic than diterpenoids and have significant medicinal effects [15, 16]. These results suggested that sesquiterpene pyridine alkaloids from *Tripterygium* could be utilized as a valuable chemical probe or a chemical moiety for the dissection of complex biological processes, discovery of unknown molecular relationships, and identification of therapeutic target molecules and pathways, which were worthy of in-depth study. The molecular mechanisms induced by sesquiterpene pyridine alkaloids from *Tripterygium* and the resulting changes in cellular phenotypes are rarely studied.

Network pharmacology is emerging as a promising strategy which is closely related to the application of multiple omics- and systems biology-based technologies [17]. It is a valuable tool for achieving a holistic view and comprehensive and systematic insights into the mechanisms of multi-ingredient medicine. Various molecular networks of complex ingredients and multilevel target-based protein and gene interactions have been constructed for predicting their functions and promoting discovery of active compounds [18]. Next, the identification of drug targets is preliminarily validated by molecular docking. After that, molecular biology experiments are conducted to further explore and accelerate the drug discovery processes.

These findings prompted us to systematically investigate the sesquiterpene pyridine alkaloids from *Tripterygium*. As a result, the greatest structural difference of 55 sesquiterpene pyridine alkaloids from *Tripterygium* lied in 2-(carboxyalkyl) nicotinic acid part possessing pyridine ring and the pharmacophore model-5 (Pm-05) was the best model that consisted of three features including being hydrogen bond acceptor (HBA), hydrogen bond donor (HBD), and hydrophobic (HY), especially hydrophobic group located in the pyridine ring. 2-(Carboxyalkyl) nicotinic acid part possessing a pyridine ring system as the pharmacologically active center and the core of structural diversity of alkaloids from *Tripterygium wilfordii* was proposed. Moreover, sesquiterpene pyridine alkaloids from *Tripterygium* were predicted to target multiple proteins and pathways and possibly participated in the treatment of Alzheimer's disease, breast cancer, Chagas disease, and NAFLD. They also had other pharmacological effects, depending on the binding interactions between pyridine rings of these compounds and active cavities of the target genes encoding PTAFR, CNR1, CNR2, FDFT1, and HSP90AA1. The elaboration of 2-(carboxyalkyl) nicotinic acid part from macrocyclic dilactone skeleton as an active group and prediction of potential targets provide a promising resource for further being utilized as a chemical moiety, lead compound, or active ingredient for future drug discovery.

## 2. Materials and Methods

### 2.1. Collection of Sesquiterpene Pyridine Alkaloids from Tripterygium

Based on related literature and the HR-MS-Database of macrocyclic dilactone skeleton alkaloids from *Tripterygium* established by our research group, data on sesquiterpene pyridine alkaloids from *Tripterygium* were extracted, and their molecular structures were converted into the standard Canonical SMILES format using PubChem database (https://pubchem.ncbi.nlm.nih.gov) and ChemSpider database (http://www.chemspider.com/), for target prediction of compounds [19, 20].

### 2.2. Pharmacophore Model Construction and Validation of Sesquiterpene Pyridine Alkaloids from Tripterygium

The ligand-based pharmacophore approach is a standard and very efficient way to conduct large virtual screening. There are multiple public sources, including OpenPHACTS, ChEMBL, and PubChem, for collecting compounds and activity data [21]. Six training set molecules from the sesquiterpene pyridine alkaloids from *Tripterygium* were taken as starting points for further ligand-based pharmacophore design and were imported in the same window of Discovery Studio software 2016 software. Then, a principal value was set at 2 to make sure that the chemical features of all the ligands were considered when hypothesis space was generated, while a MaxOmitFeat value at zero could force to map all ligand features. Before starting the pharmacophore generation process, the small molecular structures were optimized by Minimize Ligands program. The number of conformers generated for each ligand was limited to a maximum number of 200.

The Pharmacophores module in the Discovery Studio 2016 software was employed to build the pharmacophore models with qualitative common features [22]. Maximum pharmacophore hypotheses were set to 10 and the minimum interfeature distance was set at 0.5, while all the other parameters were set at default values. The generated pharmacophore models were validated by fit values of test set.

### 2.3. Direct Target Fishing for Sesquiterpene Pyridine Alkaloids from Tripterygium

The TargetNet web server (http://targetnet.scbdd.com) is an open web server that could be used for netting or predicting the binding of multiple targets for any given molecule [23]. TargetNet simultaneously constructs numerous QSAR models based on current chemogenomics data to make future predictions. Given a compound, TargetNet can provide the corresponding target genes sorted by the interactions between them in descending order. Candidate targets of sesquiterpene pyridine alkaloids from *Tripterygium* were predicted using TargetNet with default parameters. To improve the reliability of the target prediction results, only targets with a high probability score (≥0.40) were selected.

### 2.4. Analysis by GeneMANIA

GeneMANIA (http://www.genemania.org) is a user-friendly and flexible web server, for generating gene function hypotheses, analyzing gene lists, and prioritizing genes for functional assays [24, 25]. Given a query list, genes with similar function can be listed by GeneMANIA, which identifies and shows a functional relationship network according to available genomics and proteomics data. GeneMANIA also reports weights that indicate the predictive value of each selected data set for the query [24]. Six organisms are currently supported (*Arabidopsis thaliana*, *Caenorhabditis elegans*, *Drosophila melanogaster*, *Mus musculus*, *Homo sapiens,* and *Saccharomyces cerevisiae*) in GeneMANIA. The putative target genes were entered into the search bar after selecting “*Homo sapiens*” from the organism option, and the results were further collated.

### 2.5. Gene Ontology and Pathway Enrichment Analysis

The DAVID database (https://david.ncifcrf.gov/) can be utilized to thoroughly clarify and understand the functional and pathway enrichment information of a gene of interest [26]. Potential candidate targets were submitted to the DAVID database for enrichment analysis. Results of the enrichment analysis were illustrated by ClusterProfiler and ggplot2 package in R [27]. The R visualization package GOPlot was used to achieve better visualization of the relationships between genes and the selected functional categories.

### 2.6. Interaction Analysis between Putative Targets and Corresponding Diseases

The Online Mendelian Inheritance in Man (OMIM, https://omim.org/) is a knowledge source and database for human genetic diseases and related genes [28]. Each OMIM entry includes clinical synopsis, linkage analysis for candidate genes, chromosomal localization, and animal models, which has become an authoritative source of information for the study of the relationship between genes and diseases. The predicted target data were imported into the OMIM to determine the diseases related to the target. Then, the raw data were screened through manual removal of symptoms, congenital diseases, pathological processes, and animal diseases.

### 2.7. Network Construction

In order to understand the complex relationships between compounds, targets, and diseases, we used the network visualization software Cytoscape (version 3.6.0) to construct and analyze the two-layer networks (compound-target network) and three-layer networks (compound-target -disease network) [29].

### 2.8. Molecular Docking Simulation

To verify the binding affinity of candidate targets to constituent compounds of sesquiterpene pyridine alkaloids from *Tripterygium*, a molecular docking simulation was performed using the program LibDock implemented in Discovery Studio 2016. The crystal structures of the candidate targets were directly downloaded from the Protein Data Bank (PDB, http://www.pdb.org) and their resolutions varying from 1.0 to 3.5 Å were carefully examined. The higher the resolution is, the better the actual locations of separate atoms can be assigned. To evaluate the binding affinity of each candidate target to the corresponding compound, a docking score was calculated by the customizable scoring function of LibDock.

## 3. Results

### 3.1. Information Collection and Classification of Sesquiterpene Pyridine Alkaloids from Tripterygium

**Figure 1:**
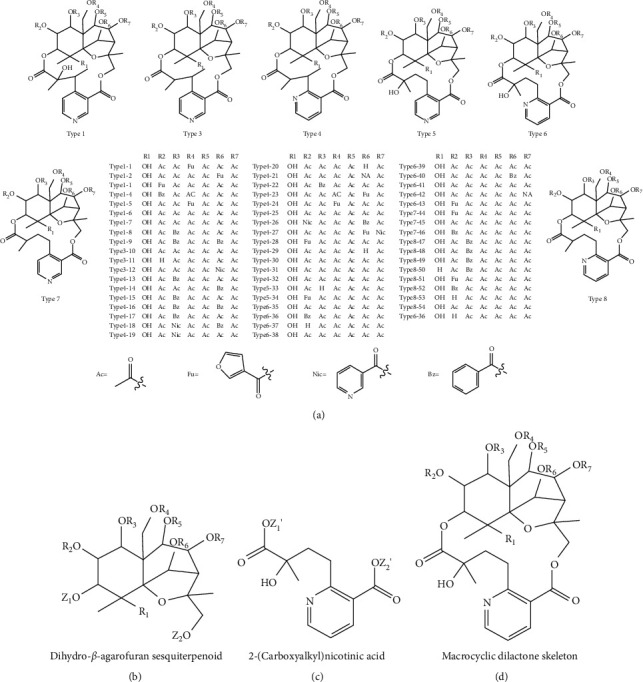
Structures of sesquiterpene pyridine alkaloids from *Tripterygium*: (a) 55 sesquiterpene pyridine alkaloids of which pyridine ring 2′, 3′ position or 3′, 4′ position are connected to macrocyclic; (b) dihydro-*β*-agarofuran sesquiterpenoid moiety; (c) 2-(carboxyalkyl) nicotinic acid moiety; (d) macrocyclic dilactone skeleton from *Tripterygium*.

A total of 55 sesquiterpene pyridine alkaloids from *Tripterygium* were collected and sorted. Detailed information consisting of compound name, molecular formula, class, and data sources is shown in Supplementary Table S1. They were divided into seven categories based on the structural differences of niacin derivatives and listed as follows: Type1, Type3, Type4, Type5, Type6, Type7, and Type8 (Figure 1). The sesquiterpene pyridine alkaloids were a large group of highly oxygenated sesquiterpenoids, all of which possessed a characteristic macrocyclic dilactone skeleton consisting of a dicarboxylic acid moiety, 2-(carboxyalkyl) nicotinic acid, and a polyoxygenated dihydro-*β*-agarofuran sesquiterpenoid (Figure 1). The sesquiterpene alkaloids were usually formed by the esterification of their hydroxy groups including acetylation, benzoylation, furanoylation, and nicotinylation. And pyridine ring 2′, 3′ position or 3′, 4′ position connected to macrocyclic were important factors of complexity of sesquiterpene pyridine alkaloids from *Tripterygium*.

### 3.2. Characteristics of Pharmacophore Model for Sesquiterpene Pyridine Alkaloids from Tripterygium

**Figure 2:**
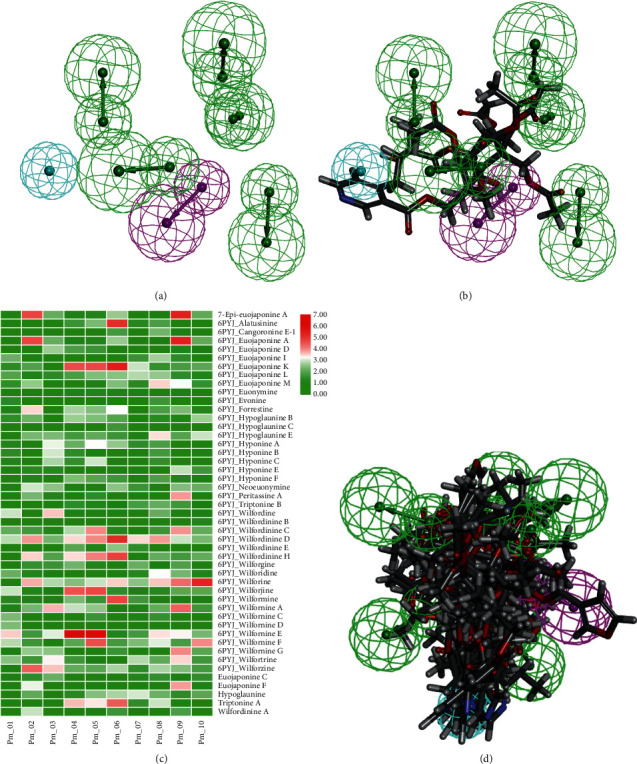
Pharmacophore models generated using six alkaloids. (a) Pharmacophore-1 (Pm_-_01) with three features, including HBA (green), HBD (pink), and HY (blue). (b) Compound wilfordinine G mapping with pharmacophore-1. (c) Heat map analysis of pharmacophore model validation. (d) Compound wilfornine E, wilforjine, euojaponine K, wilfordinine H, wilfordinine D, hyponine A, euojaponine L, hypoglaunine E, wilfordinine E, and hyponine B mapping with pharmacophore-5 (Pm_-_05).

As a continuation of the chemical studies of *Tripterygium* sesquiterpene pyridine alkaloids to mine a pharmacologically active center, pharmacophore model of alkaloids characterized by macrocyclic dilactone skeleton from *Tripterygium* was constructed. The model generation was based on the structural information of six training set molecules. Detailed information on these molecules is provided in Supplementary Table S2. A ligand-based pharmacophore model (Pm_-_01) was developed and screened using Common Feature Pharmacophore Generation (HipHop algorithm) of Discovery Studio 2016 and Fit values (Figures 2(a) and 2(b)). All the compounds of the training set shared an essential common amide functionality. In order to improve the sensitivity of the pharmacophore model, 10 pharmacophore models were validated using 41 active ligands and seven inactive ligands (Supplementary Table S2), and the best model (Pm_-_05) was selected on the basis of heat mapping results depicted by Fit values, in which the “amide” feature was replaced by a hydrogen bond acceptor (HBA) feature. The blue circle, where the pyridine ring was formed, might be an important functional feature of the sesquiterpene pyridine alkaloids (Figures 2(c) and 2(d)). Though the establishment of pharmacophore model of alkaloids containing macrocyclic dilactone skeleton from *Tripterygium*, 2-(carboxyalkyl) nicotinic acid derivatives with functional groups possessing pharmacophore features were analyzed and proposed.

### 3.3. Target Identification and Construction of Compound-Target Network for Sesquiterpene Pyridine Alkaloids from Tripterygium

**Figure 3:**
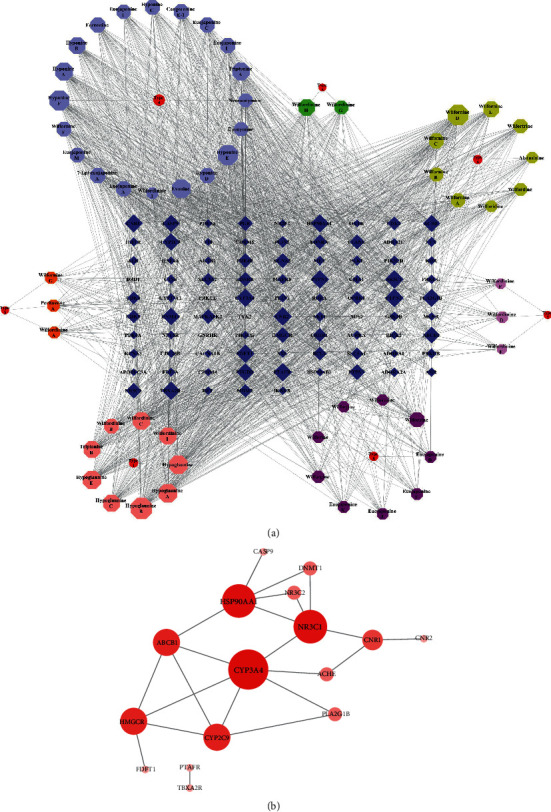
Interactions between compounds and targets for sesquiterpene pyridine alkaloids from *Tripterygium*. (a) Compound-target network for 55 sesquiterpene pyridine alkaloids. Octagons are alkaloids, and circles (red) indicated seven categories, like Type 1, Type 3, Type 4, Type 5, Type 6, Type 7, and Type 8. Diamonds (blue) are targets with the node size according to “degree” in the node size mapping. (b) Target-target interaction network for key interacting genes with the node size according to “degree” in the node size mapping.

For the purpose of target identification of alkaloids characterized by macrocyclic dilactone skeleton from *Tripterygium*, the reverse targeting strategy was firstly applied to acquire valuable targets. In the compound-target network of sesquiterpene pyridine alkaloids from *Tripterygium*, 55 compounds yielded 86 candidate target genes after removing the nonhuman genes, filtering the genes (probability < 0.4), and eliminating all duplicates. The detailed information on these targets is provided in Supplementary Table S3. Specifically, the network included 148 nodes and 1978 compound-target interactions, suggesting the presence of complex correlations among different compounds and targets (Figure 3(a)).

After the construction of a compound-target (C-T) interaction network and calculation of the four topological features, “degree,” “betweenness centrality,” “closeness centrality,” and “clustering coefficient,” 20 known targets were screened, and their detailed information is provided in Supplementary Table S4. Starting from this graph, we generated a target-target (T-T) network with the help of STRING web server (https://string-db.org/). The relationship between the targets is shown in Figure 3(b). These identified interacting genes were used for further investigation that included relationships between compounds and related diseases and their mechanism of action.

### 3.4. GeneMANIA Analysis of Putative Targets

**Figure 4:**
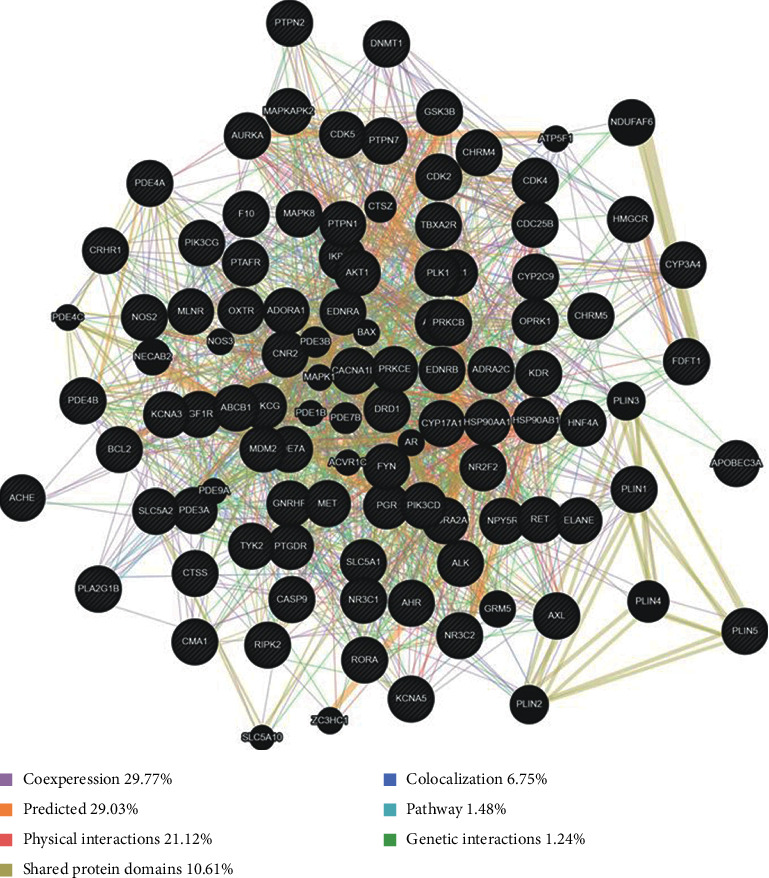
Target network of sesquiterpene pyridine alkaloids. Black nodes represented target proteins, and connecting colors indicated different correlations. Functional associations between targets were investigated using GeneMania. Genes in black circles were query terms, while the gray circles indicated genes associated with query genes.

Among the 86 key targets and their interacting proteins, 21.12% had physical interactions, 6.75% showed colocalization, and 29.77% displayed similar coexpression characteristics. Other results, including pathways and predicted and shared protein domains and genetic interactions, are shown in Figure 4.

### 3.5. GO and Pathway Analysis

**Figure 5:**
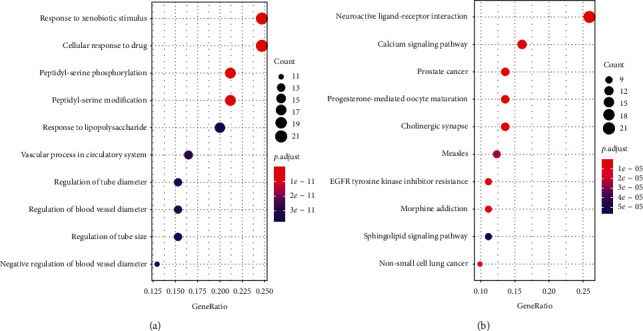
Gene ontology (GO) and pathway enrichment analysis of the putative targets. (a) GO biological function analysis (top 10). (b) KEGG enrichment analysis of the 86 hub genes (top 10).

In order to further study the 86 identified target genes, GO and KEGG enrichment analyses were conducted using DAVID database. GO enrichment analysis identified 1230 entries, which were categorized into biological processes, cellular components, and molecular function (excluding pathways that belong to the section of human diseases) to be significantly enriched with putative targets (*p* ≤ 0.05, Supplementary Table S5), in which the top four functions were used as response to xenobiotic stimulus, cellular response to drug, peptidyl-serine phosphorylation, and peptidyl-serine modification (Figure 5(a)). KEGG pathway analysis revealed that the 86 targets were assigned to 210 pathways (Supplementary Table S6), but the targets participated in 10 KEGG pathways with significant false discovery rate- (FDR-) adjusted *p*-value including neuroactive ligand-receptor interaction and pathways in cancer, which are shown in Figure 5(b).

### 3.6. Establishment of the Compound-Target-Disease Network for Sesquiterpene Pyridine Alkaloids from Tripterygium

**Figure 6:**
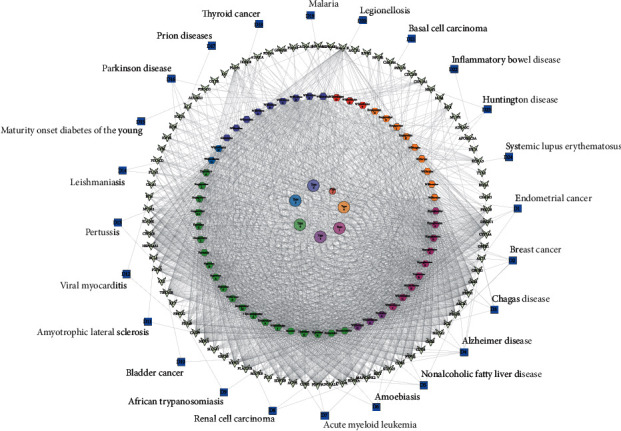
Compound-target-disease network for sesquiterpene pyridine alkaloids from *Tripterygium*. Round nodes with seven colors show seven different types of alkaloid, and arrowhead nodes represent putative targets of alkaloids. Twenty-four types of putative diseases are marked with blue squares and displayed in the outermost interaction circle.

The network map of potential targets associated with diseases was constructed, as shown in Figure 6. The network contained 172 nodes and 2044 edges (86 direct targets and 24 related diseases). The number of direct targets involved was 86 in the C-T network diagram, but only 25 corresponding targets were related to human diseases in the compound-target-disease (C-T-D) network graph. Therefore, 71 targets did not have related diseases in the network. The top eight direct targets were CASP9 (degree = 56), CYP3A4 (degree = 55), PTPN1 (degree = 55), PTAFR (degree = 55), CNR2 (degree = 55), FDFT1 (degree = 55), NR3C1 (degree = 55), and TBXA2R (degree = 55). The top four diseases were D4 (Alzheimer disease, 9), D2 (breast cancer, 6), D3 (Chagas disease, 5), and D5 (nonalcoholic fatty liver disease, 5). The detailed information on these targets and diseases is provided in Supplementary Table S7.

### 3.7. Molecular Docking Analysis

**Figure 7:**
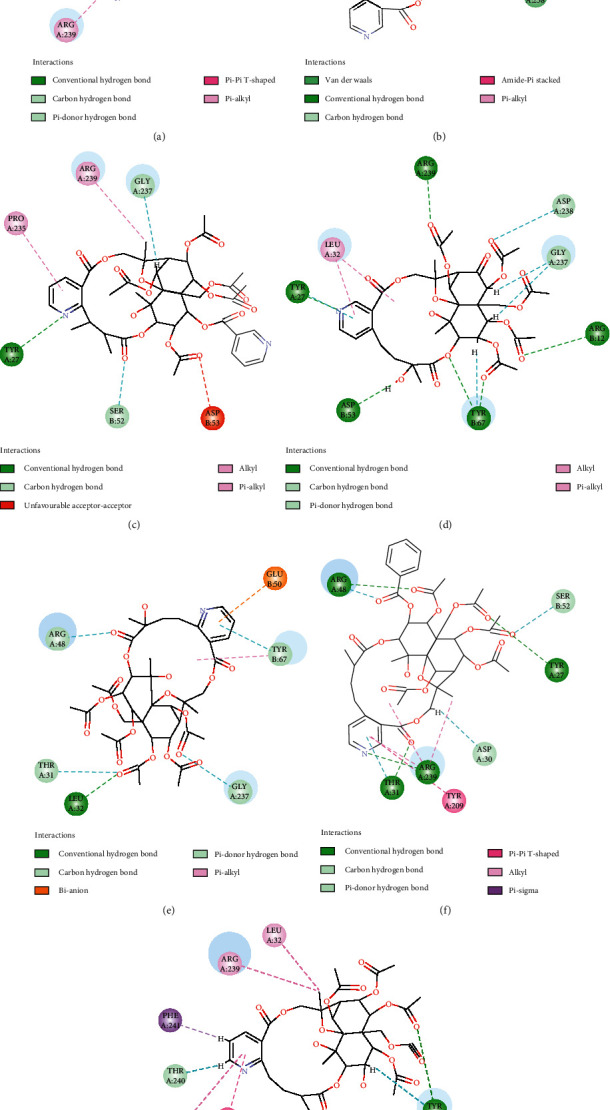
Schematic contact map of seven alkaloids and the FDFT1 (1.44 Å, PDB code 6PYJ) pocket: (a) hypoglaunine C (docking scores 112.66); (b) wilfornine G (docking scores 98.85); (c) euojaponine I (docking scores 107.11); (d) wilfordinine G (docking scores 120.87); (e) alatusinine (docking scores 97.51); (f) wilfordinine F (docking scores 109.26); (g) wilforjine (docking scores 120.19).

In order to test and verify interaction between sesquiterpene pyridine alkaloids from *Tripterygium* and their candidate targets, a molecular docking study was performed using the Libdock module implemented in Discovery Studio 2016. Molecular docking analyses on the interactions of the 55 compounds with 20 candidate targets were performed to confirm their binding abilities, and the three-dimensional (3D) structures of targets were downloaded in the Protein Data Bank (https://www.rcsb.org/). Detailed information on these targets is provided in Supplementary Table S8. Molecular docking scores indicated that the majority of 55 compounds have major interaction and binding activity with PTAFR, CNR1, CNR2, FDFT1, and HSP90AA1(Supplementary Table S9). The two-dimensional diagrams (Figure 7) displayed the interactions of seven alkaloids with the amino acid residues in the active cavity of FDFT1. Pyridine ring of hypoglaunine C could bind the Arg 239 and Asp 238 residues of FDFT1 via Pi-alkyl and hydrogen binding interactions. Pyridine ring of wilfordinine G could bind the Tyr 27 and Leu 32 residues of FDFT1 via Pi-alkyl and hydrogen binding interactions. For euojaponine I, wilfordinine F, and wilforjine, the Tyr 27, Arg 239, and Thr 240 residues were critical for their binding with FDFT1 via hydrogen binding interactions, respectively, while for alatusinine, Glu 50 residue was critical via Pi-anion binding interactions. Meanwhile, wilfornine G could only bind Arg 48, Arg 239, and Tyr 67 residues of FDFT1 via hydrogen binding interactions.

## 4. Discussion

As the representative ingredients in *Tripterygium* plants, sesquiterpene pyridine alkaloids possess various structures and many pharmacological activities, which are a research hotspot [30, 31]. However, the structure-activity relationships of sesquiterpene pyridine alkaloids and their mechanisms of action were obscure.

**Figure 8:**
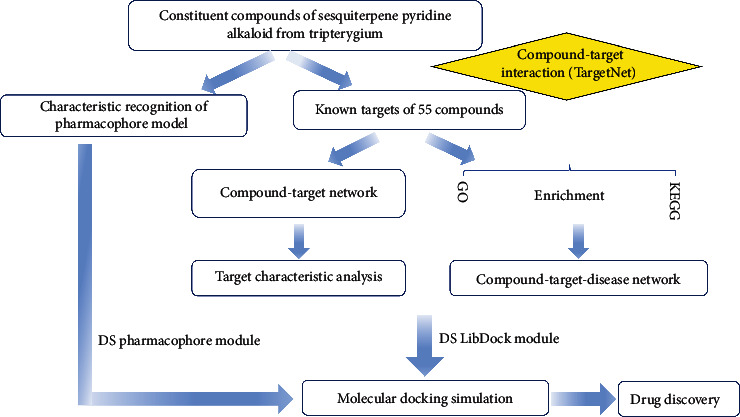
Whole workflow of this study based on network pharmacology for deciphering pharmacological mechanisms of sesquiterpene pyridine alkaloids from *Tripterygium*.

Therefore, we systematically elucidated the pharmacological actions and structural characteristics of sesquiterpene pyridine alkaloids from *Tripterygium* using computational methodologies. A schematic diagram of the analysis procedures for target gene prediction and a clear explanation of active mechanism is shown in Figure 8. In terms of structure characteristics of compounds, it was found that distinct positions connected to macrocyclic and diversity of substituted groups in the pyridine ring of alkaloid compounds led to the main structural differences. In the same way, different configuration of carboxyalkyl side chain in the 2-(carboxyalkyl) nicotinic acid moiety was an important factor in the diversity of *Tripterygium wilfordii* alkaloids. And the more significant one was the pyridine ring. As previously shown, pyridine and its derivatives (such as nicotine, nicotinic acid, and picolinic acid), among the most abundant natural N-heterocyclic compounds, were widely used in agriculture and pharmaceutical as pharmaceuticals, herbicides, and pesticides [32–34]. Thus, there has been widespread speculation that variable pharmacological functions of these sesquiterpene pyridine alkaloids might be associated with their different structures, especially pyridine ring. Meanwhile, 2-(carboxyalkyl) nicotinic acid derivatives oriented by the functional groups of *Tripterygium wilfordii* alkaloids may also become an important source of new drug development.

For further verification of functional groups of sesquiterpene pyridine alkaloids, biological effects of small molecules result from favorable interactions between the molecules and their target proteins, and chemical functionalities needed for a small molecule to block or activate its target protein could be represented as pharmacophore models [21]. Based on the establishment of the pharmacophore model in this study, pharmacophore model-5 consisted of three features including HBA, HBD, and HY, and one of these typical features was in the sesquiterpene pyridine ring, which was consistent with the analysis results, and further demonstrated that pharmacophore-5 represented the common chemical features of sesquiterpene pyridine alkaloids. Similarly, recent studies have shown that HBA, HBD, and hydrophobic region were the critical features of the hypothesis, based on wilfortrine, wilforgine, euonymine, and other CYP3A4 mechanism-based inhibitors [35]. HBA, HBD, and hydrophobic region of sesquiterpene pyridine ring possibly corresponded to the identification of compound-target interactions. These findings would promote us to deeply investigate key pharmacodynamic characteristics and build more distinct structure-activity relationship and to conduct the target fishing to forecast pharmacological action spectrum.

Target gene identification is the first step in drug discovery and elucidation of structure-activity relationship. Increasing number of active compounds or drugs have been shown to interact with multiple genes or proteins using various in silico target identification approaches [36, 37]. As listed in Supplementary Table S2, 86 potential targets of 55 sesquiterpene pyridine alkaloids were identified using computational methods. The results of GeneMANIA provided information on physical interactions, colocalization, and coexpression as well as shared protein domains and implied that the targets and their interacting proteins may have identical or similar functions. We identified that these targets participated in pathways related to cancer and inflammatory diseases. The compound-target-disease network shown in Figure 7 also revealed that the alkaloid compounds had multiple targets and exerted systematic pharmacological effects for the treatment of complex diseases by targeting multiple proteins and pathways, such as Alzheimer's disease, breast cancer, and systemic lupus erythematosus.

In addition, the topological analysis of compound-target network yielded the key targets as follows: CYP3A4, PTPN1, PTAFR, CNR2, PTPN2, FDFT1, NR3C1, TBXA2R, CNR1, PLA2G1B, HSP90AA1, PTGDR2, DNMT1, CYP2C9, CASP9, ABCB1, ACHE, NR3C2, PTPN7, and HMGCR. Among them, PTAFR located on plasma membrane was reported to be a chemotactic phospholipid mediator that possessed potent inflammatory, smooth-muscle contractile and hypotensive activities [38, 39]. ABCB1 has been considered as energy-dependent efflux pump responsible for decreased drug accumulation in multidrug-resistant cells [40]. And CYP3A4, as the most abundant CYPs in human liver, which had greater binding activities with wilfortrine when compared with wilforgine and euonymine, was involved in the metabolism and response of about 50% of all prescribed drugs [35, 41]. These results closely matched the findings from GO and KEGG analyses.

Under the premise of obtaining significantly functional groups and potential targets, 55 compounds mapped on all of the pharmacophoric features present in pharmacophore model-5 were finally used in a molecular docking study. Importantly, molecular docking results showed the molecular docking relationship with LibDock Score value between 90 and 150 points accounted for 80 pairs (75.00%) and also confirmed that the target genes PTAFR, CNR1, CNR2, FDFT1, and HSP90AA1 could better combine with most sesquiterpene pyridine alkaloids through the binding interactions between amino acid residues and pyridine rings and were mainly focused on NLRP3 inflammasome activation, IL1B and IL18 secretion, progression of type-2 diabetes, enzymes of the sterol biosynthesis pathway, and drug resistance [42–44]. We speculated that these potential proteins might participate in suppressing inflammation and related diseases and should be followed up with more interest. Furthermore, interactions of these alkaloids with the amino acid residues in the active cavity of key targets mainly focused on pyridine ring, which well corroborated with the pharmacophore model and prompted us to note that 2-(carboxyalkyl) nicotinic acid derivatives assisted in discovering new potential leads with the treatment of inflammatory disease.

In summary, we analyzed and screened the relevant pharmacological laws, important targets, and the binding sites of niacin derivatives that exert their pharmacodynamics. Sesquiterpene pyridine alkaloids from *Tripterygium* are promising compounds for the development of safe and effective multitargeted anticancer or anti-inflammatory medicines. This study also provided novel insights into the experimental directions and challenges for the follow-up study on pharmacodynamic material basis of sesquiterpene pyridine alkaloids from *Tripterygium*. However, these findings needed to be further substantiated under the experimental system.

## Data Availability

All data that support the findings of this study are included in this published article.

## Abbreviations

GO: Gene ontology
KEGG: Kyoto encyclopedia of genes and genomes
DS: Discovery studio software.

## Supplementary Materials

Supplementary Materials Supplementary information is available for this paper and listed as follows. Supplementary Table S1: sesquiterpene pyridine alkaloids from *Tripterygium* classified by structural differences of niacin derivatives. Supplementary Table S2: molecules of pharmacophore model construction and validation for sesquiterpene pyridine alkaloids from *Tripterygium*. Supplementary Table S3: putative targets of sesquiterpene pyridine alkaloids from *Tripterygium*. Supplementary Table S4: topological parameters of key targets for sesquiterpene pyridine alkaloids from *Tripterygium*. Supplementary Table S5: GO enrichment analysis of targets. Supplementary Table S6: KEGG enrichment analysis of targets. Supplementary Table S7: putative diseases of targets for sesquiterpene pyridine alkaloids from *Tripterygium*. Supplementary Table S8: information of target proteins for molecular docking. Supplementary Table S9: molecular docking results of compound-target pairs (
