# Supplementary material for: The Exploration of Novel Pharmacophore Characteristics and Multidirectional Elucidation of Structure-Activity Relationship and Mechanism of Sesquiterpene Pyridine Alkaloids from Tripterygium Based on Computational Approaches
Source: Evid Based Complement Alternat Med. 2021 Mar 24;2021:6676470. doi: 10.1155/2021/6676470 (PMC8012133; doi:10.1155/2021/6676470)
Supplement: Supplementary Materials — Supplementary information is available for this paper and listed as follows. Supplementary Table S1: sesquiterpene pyridine alkaloids from Tripterygium classified by structural differences of niacin derivatives. Supplementary Table S2: molecules of pharmacophore model construction and validation for sesquiterpene pyridine alkaloids from Tripterygium. Supplementary Table S3: putative targets of sesquiterpene pyridine alkaloids from Tripterygium. Supplementary Table S4: topological parameters of key targets for sesquiterpene pyridine alkaloids from Tripterygium. Supplementary Table S5: GO enrichment analysis of targets. Supplementary Table S6: KEGG enrichment analysis of targets. Supplementary Table S7: putative diseases of targets for sesquiterpene pyridine alkaloids from Tripterygium. Supplementary Table S8: information of target proteins for molecular docking. Supplementary Table S9: molecular docking results of compound-target pairs ( [file 6676470.f1.zip › 6676470.f1/[Manuscript] Supplementary Table [S1].docx]

**Supplementary Table S1 Sesquiterpene pyridine alkaloids from Tripterygium classified by structural differences of niacin derivatives.**

| Num. | Compound | Molecular formula | Class | Reference |
| --- | --- | --- | --- | --- |
| 1 | Hypoglaunine | C_41_H_47_NO_20_ | Type 1 | 30 |
| 2 | Hypoglaunine A | C_41_H_47_NO_20_ | Type 1 | 14 |
| 3 | Hypoglaunine B | C_41_H_47_NO_20_ | Type 1 | 31 |
| 4 | Hypoglaunine C | C_43_H_49_NO_19_ | Type 1 | 32 |
| 5 | Hypoglaunine E | C_39_H_45_NO_19_ | Type 1 | 14 |
| 6 | Triptonine B | C_46_H_50_NO_22_ | Type 1 | 33 |
| 7 | Wilfordinine B | C_38_H_47_NO_19_ | Type 1 | 30 |
| 8 | Wilfordinine C | C_43_H_49_NO_19_ | Type 1 | 30 |
| 9 | Wilfordinine I | C_48_H_51_NO_19_ | Type 1 | 32 |
| 10 | Peritassine A | C_38_H_47_NO_18_ | Type 3 | 33 |
| 11 | Wilfordinine A | C_36_H_45_NO_17_ | Type 3 | 14 |
| 12 | Wilfornine G | C_42_H_48_N_2_O_18_ | Type 3 | 34 |
| 13 | 7-Epi-euojaponine A | C_41_H_47_NO_17_ | Type 4 | 35 |
| 14 | Cangoronine E-1 | C_43_H_49_NO_18_ | Type 4 | 36 |
| 15 | Euojaponine A | C_41_H_47_NO_17_ | Type 4 | 35 |
| 16 | Euojaponine C | C_46_H_49_NO_17_ | Type 4 | 37 |
| 17 | Euojaponine I | C_42_H_48_N_2_O_18_ | Type 4 | Database |
| 18 | Euojaponine L | C_45_H_48_N_2_O_17_ | Type 4 | Database |
| 19 | Euojaponine M | C_40_H_46_N_2_O_17_ | Type 4 | Database |
| 20 | Euonymine | C_38_H_47_NO_18_ | Type 4 | 36 |
| 21 | Evonine | C_36_H_43_NO_17_ | Type 4 | 38 |
| 22 | Forrestine | C_41_H_47_NO_19_ | Type 4 | 39 |
| 23 | Hyponine A | C_41_H_47_NO_19_ | Type 4 | Database |
| 24 | Hyponine B | C_41_H_47_NO_19_ | Type 4 | 40 |
| 25 | Hyponine C | C_43_H_49_NO_18_ | Type 4 | 35 |
| 26 | Hyponine D | C_47_H_50_N_2_O_18_ | Type 4 | 35 |
| 27 | Hyponine E | C_45_H_48_N_2_O_19_ | Type 4 | Database |
| 28 | Hyponine F | C_41_H_47_NO_19_ | Type 4 | Database |
| 29 | Neoeuonymine | C_36_H_45_NO_17_ | Type 4 | 36 |
| 30 | Triptonine A | C_45_H_55_NO_21_ | Type 4 | 40 |
| 31 | Wilfordinine J | C_36_H_45_NO_17_ | Type 4 | 32 |
| 32 | Wilfornine F | C_41_H_47_NO_17_ | Type 4 | 35 |
| 33 | Wilfordinine G | C_36_H_43_NO_16_ | Type 5 | 41 |
| 34 | Wilfordinine H | C_40_H_47_NO_19_ | Type 5 | 41 |
| 35 | Alatusinine | C_38_H_47_NO_19_ | Type 6 | 35 |
| 36 | Wilfordine | C_43_H_49_NO_19_ | Type 6 | 42 |
| 37 | Wilforidine | C_36_H_45_NO_18_ | Type 6 | Database |
| 38 | Wilfornine A | C_45_H_51_NO_20_ | Type 6 | 16 |
| 39 | Wilfornine B | C_43_H_49_NO_19_ | Type 6 | 32 |
| 40 | Wilfornine C | C_50_H_53_NO_20_ | Type 6 | 32 |
| 41 | Wilfornine D | C_43_H_49_NO_21_ | Type 6 | 16 |
| 42 | Wilfornine E | C_36_H_43_NO_18_ | Type 6 | 32 |
| 43 | Wilfortrine | C_41_H_47_NO_20_ | Type 6 | 42 |
| 44 | Wilfordinine D | C_41_H_47_NO_19_ | Type 7 | 41 |
| 45 | Wilfordinine E | C_38_H_47_NO_18_ | Type 7 | 14 |
| 46 | Wilfordinine F | C_43_H_49_NO_18_ | Type 7 | 41 |
| 47 | Euojaponine D | C_41_H_47_NO_17_ | Type 8 | Database |
| 48 | Euojaponine F | C_43_H_49_NO_18_ | Type 8 | 39 |
| 49 | Euojaponine J | C_41_H_47_NO_16_ | Type 8 | Database |
| 50 | Euojaponine K | C_41_H_47_NO_17_ | Type 8 | Database |
| 51 | Wilforgine | C_41_H_47_NO_19_ | Type 8 | 42 |
| 52 | Wilforine | C_43_H_49_NO_18_ | Type 8 | 42 |
| 53 | Wilforjine | C_36_H_45_NO_17_ | Type 8 | 35 |
| 54 | Wilformine | C_38_H_47_NO_18_ | Type 8 | 42 |
| 55 | Wilforzine | C_41_H_47_NO_17_ | Type 8 | 43 |

“Database” represented that related information on sesquiterpene pyridine alkaloids from Tripterygium were obtained from HR-MS-Database of macrocyclic dilactone skeleton alkaloids from Tripterygium established by our research group.
