# Supplementary material for: The Exploration of Novel Pharmacophore Characteristics and Multidirectional Elucidation of Structure-Activity Relationship and Mechanism of Sesquiterpene Pyridine Alkaloids from Tripterygium Based on Computational Approaches
Source: Evid Based Complement Alternat Med. 2021 Mar 24;2021:6676470. doi: 10.1155/2021/6676470 (PMC8012133; doi:10.1155/2021/6676470)
Supplement: Supplementary Materials — Supplementary information is available for this paper and listed as follows. Supplementary Table S1: sesquiterpene pyridine alkaloids from Tripterygium classified by structural differences of niacin derivatives. Supplementary Table S2: molecules of pharmacophore model construction and validation for sesquiterpene pyridine alkaloids from Tripterygium. Supplementary Table S3: putative targets of sesquiterpene pyridine alkaloids from Tripterygium. Supplementary Table S4: topological parameters of key targets for sesquiterpene pyridine alkaloids from Tripterygium. Supplementary Table S5: GO enrichment analysis of targets. Supplementary Table S6: KEGG enrichment analysis of targets. Supplementary Table S7: putative diseases of targets for sesquiterpene pyridine alkaloids from Tripterygium. Supplementary Table S8: information of target proteins for molecular docking. Supplementary Table S9: molecular docking results of compound-target pairs ( [file 6676470.f1.zip › 6676470.f1/[Manuscript] Supplementary Table [S2].docx]

**Supplementary Table S2 Molecules of pharmacophore model construction and validation for sesquiterpene pyridine alkaloids from Tripterygium.**

| Num. | Name | Active or inactive |
| --- | --- | --- |
| Training set molecule 1 | Wilfordinine I | active |
| Training set molecule 2 | Wilfordinine J | active |
| Training set molecule 3 | Wilfordinine G | active |
| Training set molecule 4 | Wilfornine B | active |
| Training set molecule 5 | Wilfordinine F | active |
| Training set molecule 6 | Euojaponine J | active |
| Decoy set molecule 1 | Hypoglaunine B | active |
| Decoy set molecule 2 | Hypoglaunine C | active |
| Decoy set molecule 3 | Hypoglaunine E | active |
| Decoy set molecule 4 | Triptonine B | active |
| Decoy set molecule 5 | Wilfordinine B | active |
| Decoy set molecule 6 | Wilfordinine C | active |
| Decoy set molecule 7 | Peritassine A | active |
| Decoy set molecule 8 | Wilfornine G | active |
| Decoy set molecule 9 | Cangoronine E-1 | active |
| Decoy set molecule 10 | Euojaponine A | active |
| Decoy set molecule 11 | Euojaponine I | active |
| Decoy set molecule 12 | Euojaponine L | active |
| Decoy set molecule 13 | Euojaponine M | active |
| Decoy set molecule 14 | Euonymine | active |
| Decoy set molecule 15 | Evonine | active |
| Decoy set molecule 16 | Forrestine | active |
| Decoy set molecule 17 | Hyponine A | active |
| Decoy set molecule 18 | Hyponine B | active |
| Decoy set molecule 19 | Hyponine C | active |
| Decoy set molecule 20 | Hyponine E | active |
| Decoy set molecule 21 | Hyponine F | active |
| Decoy set molecule 22 | Neoeuonymine | active |
| Decoy set molecule 23 | Wilfornine F | active |
| Decoy set molecule 24 | Wilfordinine H | active |
| Decoy set molecule 25 | Alatusinine | active |
| Decoy set molecule 26 | Wilfordine | active |
| Decoy set molecule 27 | Wilforidine | active |
| Decoy set molecule 28 | Wilfornine A | active |
| Decoy set molecule 29 | Wilfornine C | active |
| Decoy set molecule 30 | Wilfornine D | active |
| Decoy set molecule 31 | Wilfornine E | active |
| Decoy set molecule 32 | Wilfortrine | active |
| Decoy set molecule 33 | Wilfordinine D | active |
| Decoy set molecule 34 | Wilfordinine E | active |
| Decoy set molecule 35 | Euojaponine D | active |
| Decoy set molecule 36 | Euojaponine K | active |
| Decoy set molecule 37 | Wilforgine | active |
| Decoy set molecule 38 | Wilforine | active |
| Decoy set molecule 39 | Wilforjine | active |
| Decoy set molecule 40 | Wilformine | active |
| Decoy set molecule 41 | Wilforzine | active |
| Decoy set molecule 42 | Hypoglaunine | inactive |
| Decoy set molecule 43 | Hypoglaunine A | inactive |
| Decoy set molecule 44 | Wilfordinine A | inactive |
| Decoy set molecule 45 | 7-Epi-euojaponine A | inactive |
| Decoy set molecule 46 | Euojaponine C | inactive |
| Decoy set molecule 47 | Triptonine A | inactive |
| Decoy set molecule 48 | Euojaponine F | inactive |
