# Supplementary material for: The Exploration of Novel Pharmacophore Characteristics and Multidirectional Elucidation of Structure-Activity Relationship and Mechanism of Sesquiterpene Pyridine Alkaloids from Tripterygium Based on Computational Approaches
Source: Evid Based Complement Alternat Med. 2021 Mar 24;2021:6676470. doi: 10.1155/2021/6676470 (PMC8012133; doi:10.1155/2021/6676470)
Supplement: Supplementary Materials — Supplementary information is available for this paper and listed as follows. Supplementary Table S1: sesquiterpene pyridine alkaloids from Tripterygium classified by structural differences of niacin derivatives. Supplementary Table S2: molecules of pharmacophore model construction and validation for sesquiterpene pyridine alkaloids from Tripterygium. Supplementary Table S3: putative targets of sesquiterpene pyridine alkaloids from Tripterygium. Supplementary Table S4: topological parameters of key targets for sesquiterpene pyridine alkaloids from Tripterygium. Supplementary Table S5: GO enrichment analysis of targets. Supplementary Table S6: KEGG enrichment analysis of targets. Supplementary Table S7: putative diseases of targets for sesquiterpene pyridine alkaloids from Tripterygium. Supplementary Table S8: information of target proteins for molecular docking. Supplementary Table S9: molecular docking results of compound-target pairs ( [file 6676470.f1.zip › 6676470.f1/[Manuscript] Supplementary Table [S3].docx]

**Supplementary Table S3 Putative targets of** **sesquiterpene pyridine alkaloids from Tripterygium.**

| Num. | Gene ID | Gene symbol | Gene name |
| --- | --- | --- | --- |
| 1 | 1786 | DNMT1 | DNA methyltransferase 1 |
| 2 | 1268 | CNR1 | cannabinoid receptor 1 |
| 3 | 5770 | PTPN1 | protein tyrosine phosphatase non-receptor type 1 |
| 4 | 5243 | ABCB1 | ATP binding cassette subfamily B member 1 |
| 5 | 5724 | PTAFR | platelet activating factor receptor |
| 6 | 3156 | HMGCR | 3-hydroxy-3-methylglutaryl-CoA reductase |
| 7 | 1269 | CNR2 | cannabinoid receptor 2 |
| 8 | 5771 | PTPN2 | protein tyrosine phosphatase non-receptor type 2 |
| 9 | 4986 | OPRK1 | opioid receptor kappa 1 |
| 10 | 3741 | KCNA5 | potassium voltage-gated channel subfamily A member 5 |
| 11 | 9261 | MAPKAPK2 | MAPK activated protein kinase 2 |
| 12 | 1909 | EDNRA | endothelin receptor type A |
| 13 | 5729 | PTGDR | prostaglandin D2 receptor |
| 14 | 2222 | FDFT1 | farnesyl-diphosphate farnesyltransferase 1 |
| 15 | 43 | ACHE | acetylcholinesterase (Cartwright blood group) |
| 16 | 1576 | CYP3A4 | cytochrome P450 family 3 subfamily A member 4 |
| 17 | 5319 | PLA2G1B | phospholipase A2 group IB |
| 18 | 4306 | NR3C2 | nuclear receptor subfamily 3 group C member 2 |
| 19 | 2908 | NR3C1 | nuclear receptor subfamily 3 group C member 1 |
| 20 | 5142 | PDE4B | phosphodiesterase 4B |
| 21 | 6915 | TBXA2R | thromboxane A2 receptor |
| 22 | 5778 | PTPN7 | protein tyrosine phosphatase non-receptor type 7 |
| 23 | 3320 | HSP90AA1 | heat shock protein 90 alpha family class A member 1 |
| 24 | 1559 | CYP2C9 | cytochrome P450 family 2 subfamily C member 9 |
| 25 | 5241 | PGR | progesterone receptor |
| 26 | 1910 | EDNRB | endothelin receptor type B |
| 27 | 2862 | MLNR | motilin receptor |
| 28 | 440503 | PLIN5 | perilipin 5 |
| 29 | 1991 | ELANE | elastase, neutrophil expressed |
| 30 | 842 | CASP9 | caspase 9 |
| 31 | 994 | CDC25B | cell division cycle 25B |
| 32 | 4889 | NPY5R | neuropeptide Y receptor Y5 |
| 33 | 1586 | CYP17A1 | cytochrome P450 family 17 subfamily A member 1 |
| 34 | 7026 | NR2F2 | nuclear receptor subfamily 2 group F member 2 |
| 35 | 4843 | NOS2 | nitric oxide synthase 2 |
| 36 | 152 | ADRA2C | adrenoceptor alpha 2C |
| 37 | 1394 | CRHR1 | corticotropin releasing hormone receptor 1 |
| 38 | 6524 | SLC5A2 | solute carrier family 5 member 2 |
| 39 | 1020 | CDK5 | cyclin dependent kinase 5 |
| 40 | 6523 | SLC5A1 | solute carrier family 5 member 1 |
| 41 | 596 | BCL2 | BCL2 apoptosis regulator |
| 42 | 4193 | MDM2 | MDM2 proto-oncogene |
| 43 | 1019 | CDK4 | cyclin dependent kinase 4 |
| 44 | 1017 | CDK2 | cyclin dependent kinase 2 |
| 45 | 6095 | RORA | RAR related orphan receptor A |
| 46 | 3738 | KCNA3 | potassium voltage-gated channel subfamily A member 3 |
| 47 | 8767 | RIPK2 | receptor interacting serine/threonine kinase 2 |
| 48 | 207 | AKT1 | AKT serine/threonine kinase 1 |
| 49 | 238 | ALK | ALK receptor tyrosine kinase |
| 50 | 134 | ADORA1 | adenosine A1 receptor |
| 51 | 1520 | CTSS | cathepsin S |
| 52 | 5150 | PDE7A | phosphodiesterase 7A |
| 53 | 3480 | IGF1R | insulin like growth factor 1 receptor |
| 54 | 5294 | PIK3CG | phosphatidylinositol-4,5-bisphosphate 3-kinase catalytic subunit gamma |
| 55 | 4233 | MET | MET proto-oncogene, receptor tyrosine kinase |
| 56 | 135 | ADORA2A | adenosine A2a receptor |
| 57 | 5347 | PLK1 | polo like kinase 1 |
| 58 | 5599 | MAPK8 | mitogen-activated protein kinase 8 |
| 59 | 2932 | GSK3B | glycogen synthase kinase 3 beta |
| 60 | 5139 | PDE3A | phosphodiesterase 3A |
| 61 | 3326 | HSP90AB1 | heat shock protein 90 alpha family class B member 1 |
| 62 | 3551 | IKBKB | inhibitor of nuclear factor kappa B kinase subunit beta |
| 63 | 5579 | PRKCB | protein kinase C beta |
| 64 | 6790 | AURKA | aurora kinase A |
| 65 | 5293 | PIK3CD | phosphatidylinositol-4,5-bisphosphate 3-kinase catalytic subunit delta |
| 66 | 2534 | FYN | FYN proto-oncogene, Src family tyrosine kinase |
| 67 | 5346 | PLIN1 | perilipin 1 |
| 68 | 5979 | RET | ret proto-oncogene |
| 69 | 3172 | HNF4A | hepatocyte nuclear factor 4 alpha |
| 70 | 196 | AHR | aryl hydrocarbon receptor |
| 71 | 5021 | OXTR | oxytocin receptor |
| 72 | 1215 | CMA1 | chymase 1 |
| 73 | 2159 | F10 | coagulation factor X |
| 74 | 200315 | APOBEC3A | apolipoprotein B mRNA editing enzyme catalytic subunit 3A |
| 75 | 1133 | CHRM5 | cholinergic receptor muscarinic 5 |
| 76 | 5582 | PRKCG | protein kinase C gamma |
| 77 | 1132 | CHRM4 | cholinergic receptor muscarinic 4 |
| 78 | 558 | AXL | AXL receptor tyrosine kinase |
| 79 | 5141 | PDE4A | phosphodiesterase 4A |
| 80 | 1812 | DRD1 | dopamine receptor D1 |
| 81 | 2798 | GNRHR | gonadotropin releasing hormone receptor |
| 82 | 774 | CACNA1B | calcium voltage-gated channel subunit alpha1 B |
| 83 | 185 | AGTR1 | angiotensin II receptor type 1 |
| 84 | 5581 | PRKCE | protein kinase C epsilon |
| 85 | 7297 | TYK2 | tyrosine kinase 2 |
| 86 | 3791 | KDR | vascular endothelial growth factor receptor 2 |
