# Supplementary material for: The Exploration of Novel Pharmacophore Characteristics and Multidirectional Elucidation of Structure-Activity Relationship and Mechanism of Sesquiterpene Pyridine Alkaloids from Tripterygium Based on Computational Approaches
Source: Evid Based Complement Alternat Med. 2021 Mar 24;2021:6676470. doi: 10.1155/2021/6676470 (PMC8012133; doi:10.1155/2021/6676470)
Supplement: Supplementary Materials — Supplementary information is available for this paper and listed as follows. Supplementary Table S1: sesquiterpene pyridine alkaloids from Tripterygium classified by structural differences of niacin derivatives. Supplementary Table S2: molecules of pharmacophore model construction and validation for sesquiterpene pyridine alkaloids from Tripterygium. Supplementary Table S3: putative targets of sesquiterpene pyridine alkaloids from Tripterygium. Supplementary Table S4: topological parameters of key targets for sesquiterpene pyridine alkaloids from Tripterygium. Supplementary Table S5: GO enrichment analysis of targets. Supplementary Table S6: KEGG enrichment analysis of targets. Supplementary Table S7: putative diseases of targets for sesquiterpene pyridine alkaloids from Tripterygium. Supplementary Table S8: information of target proteins for molecular docking. Supplementary Table S9: molecular docking results of compound-target pairs ( [file 6676470.f1.zip › 6676470.f1/[Manuscript] Supplementary Table [S4].docx]

**Supplementary Table S4 Topological parameters of key targets for sesquiterpene pyridine alkaloids from Tripterygium.**

| Gene name | Degree | Betweenness centrality | Closeness centrality | Clustering coefficient |
| --- | --- | --- | --- | --- |
| TBXA2R | 55 | 0.024 | 0.615 | 0 |
| NR3C1 | 55 | 0.024 | 0.615 | 0 |
| FDFT1 | 55 | 0.024 | 0.615 | 0 |
| PTPN2 | 55 | 0.024 | 0.615 | 0 |
| CNR2 | 55 | 0.024 | 0.615 | 0 |
| PTAFR | 55 | 0.024 | 0.615 | 0 |
| PTPN1 | 55 | 0.024 | 0.615 | 0 |
| CYP3A4 | 55 | 0.024 | 0.615 | 0 |
| HSP90AA1 | 54 | 0.022 | 0.610 | 0 |
| PLA2G1B | 54 | 0.022 | 0.610 | 0 |
| CNR1 | 54 | 0.022 | 0.610 | 0 |
| PTGDR2 | 52 | 0.020 | 0.595 | 0 |
| DNMT1 | 51 | 0.018 | 0.595 | 0 |
| CYP2C9 | 50 | 0.017 | 0.586 | 0 |
| CASP9 | 49 | 0.017 | 0.586 | 0 |
| ABCB1 | 47 | 0.017 | 0.572 | 0 |
| ACHE | 49 | 0.016 | 0.576 | 0 |
| NR3C2 | 42 | 0.011 | 0.542 | 0 |
| PTPN7 | 43 | 0.011 | 0.546 | 0 |
| HMGCR | 41 | 0.010 | 0.535 | 0 |
