# Supplementary material for: The Exploration of Novel Pharmacophore Characteristics and Multidirectional Elucidation of Structure-Activity Relationship and Mechanism of Sesquiterpene Pyridine Alkaloids from Tripterygium Based on Computational Approaches
Source: Evid Based Complement Alternat Med. 2021 Mar 24;2021:6676470. doi: 10.1155/2021/6676470 (PMC8012133; doi:10.1155/2021/6676470)
Supplement: Supplementary Materials — Supplementary information is available for this paper and listed as follows. Supplementary Table S1: sesquiterpene pyridine alkaloids from Tripterygium classified by structural differences of niacin derivatives. Supplementary Table S2: molecules of pharmacophore model construction and validation for sesquiterpene pyridine alkaloids from Tripterygium. Supplementary Table S3: putative targets of sesquiterpene pyridine alkaloids from Tripterygium. Supplementary Table S4: topological parameters of key targets for sesquiterpene pyridine alkaloids from Tripterygium. Supplementary Table S5: GO enrichment analysis of targets. Supplementary Table S6: KEGG enrichment analysis of targets. Supplementary Table S7: putative diseases of targets for sesquiterpene pyridine alkaloids from Tripterygium. Supplementary Table S8: information of target proteins for molecular docking. Supplementary Table S9: molecular docking results of compound-target pairs ( [file 6676470.f1.zip › 6676470.f1/[Manuscript] Supplementary Table [S7].docx]

**Supplementary Table S7 Putative diseases of targets for sesquiterpene pyridine alkaloids from Tripterygium.**

| Target gene | Putative disease | Disease number |
| --- | --- | --- |
| CASP9 | Endometrial cancer | D1 |
| AKT1 | Endometrial cancer | D1 |
| GSK3B | Endometrial cancer | D1 |
| PIK3CD | Endometrial cancer | D1 |
| PGR | Breast cancer | D2 |
| CDK4 | Breast cancer | D2 |
| AKT1 | Breast cancer | D2 |
| IGF1R | Breast cancer | D2 |
| GSK3B | Breast cancer | D2 |
| PIK3CD | Breast cancer | D2 |
| NOS2 | Chagas disease (American trypanosomiasis) | D3 |
| AKT1 | Chagas disease (American trypanosomiasis) | D3 |
| MAPK8 | Chagas disease (American trypanosomiasis) | D3 |
| IKBKB | Chagas disease (American trypanosomiasis) | D3 |
| PIK3CD | Chagas disease (American trypanosomiasis) | D3 |
| CASP9 | Alzheimer disease | D4 |
| NOS2 | Alzheimer disease | D4 |
| CDK5 | Alzheimer disease | D4 |
| AKT1 | Alzheimer disease | D4 |
| MAPK8 | Alzheimer disease | D4 |
| GSK3B | Alzheimer disease | D4 |
| IKBKB | Alzheimer disease | D4 |
| PIK3CD | Alzheimer disease | D4 |
| CHRM5 | Alzheimer disease | D4 |
| AKT1 | Non-alcoholic fatty liver disease (NAFLD) | D5 |
| MAPK8 | Non-alcoholic fatty liver disease (NAFLD) | D5 |
| GSK3B | Non-alcoholic fatty liver disease (NAFLD) | D5 |
| IKBKB | Non-alcoholic fatty liver disease (NAFLD) | D5 |
| PIK3CD | Non-alcoholic fatty liver disease (NAFLD) | D5 |
| NOS2 | Amoebiasis | D6 |
| PRKCB | Amoebiasis | D6 |
| PIK3CD | Amoebiasis | D6 |
| PRKCG | Amoebiasis | D6 |
| AKT1 | Acute myeloid leukemia | D7 |
| IKBKB | Acute myeloid leukemia | D7 |
| PIK3CD | Acute myeloid leukemia | D7 |
| AKT1 | Renal cell carcinoma | D8 |
| MET | Renal cell carcinoma | D8 |
| PIK3CD | Renal cell carcinoma | D8 |
| PRKCB | African trypanosomiasis | D9 |
| PRKCG | African trypanosomiasis | D9 |
| MDM2 | Bladder cancer | D10 |
| CDK4 | Bladder cancer | D10 |
| CASP9 | Amyotrophic lateral sclerosis (ALS) | D11 |
| BCL2 | Amyotrophic lateral sclerosis (ALS) | D11 |
| CASP9 | Viral myocarditis | D12 |
| FYN | Viral myocarditis | D12 |
| NOS2 | Pertussis | D13 |
| MAPK8 | Pertussis | D13 |
| NOS2 | Leishmaniasis | D14 |
| PRKCB | Leishmaniasis | D14 |
| HNF4A | Maturity onset diabetes of the young | D15 |
| CASP9 | Parkinson disease | D16 |
| ADORA2A | Parkinson disease | D16 |
| MAPK8 | Parkinson disease | D16 |
| DRD1 | Parkinson disease | D16 |
| FYN | Prion diseases | D17 |
| RET | Thyroid cancer | D18 |
| MET | Malaria | D19 |
| CASP9 | Legionellosis | D20 |
| GSK3B | Basal cell carcinoma | D21 |
| RORA | Inflammatory bowel disease (IBD) | D22 |
| CASP9 | Huntington disease | D23 |
| MAPK8 | Huntington disease | D23 |
| CACNA1B | Huntington disease | D23 |
| ELANE | Systemic lupus erythematosus | D24 |
