# Supplementary material for: The Exploration of Novel Pharmacophore Characteristics and Multidirectional Elucidation of Structure-Activity Relationship and Mechanism of Sesquiterpene Pyridine Alkaloids from Tripterygium Based on Computational Approaches
Source: Evid Based Complement Alternat Med. 2021 Mar 24;2021:6676470. doi: 10.1155/2021/6676470 (PMC8012133; doi:10.1155/2021/6676470)
Supplement: Supplementary Materials — Supplementary information is available for this paper and listed as follows. Supplementary Table S1: sesquiterpene pyridine alkaloids from Tripterygium classified by structural differences of niacin derivatives. Supplementary Table S2: molecules of pharmacophore model construction and validation for sesquiterpene pyridine alkaloids from Tripterygium. Supplementary Table S3: putative targets of sesquiterpene pyridine alkaloids from Tripterygium. Supplementary Table S4: topological parameters of key targets for sesquiterpene pyridine alkaloids from Tripterygium. Supplementary Table S5: GO enrichment analysis of targets. Supplementary Table S6: KEGG enrichment analysis of targets. Supplementary Table S7: putative diseases of targets for sesquiterpene pyridine alkaloids from Tripterygium. Supplementary Table S8: information of target proteins for molecular docking. Supplementary Table S9: molecular docking results of compound-target pairs ( [file 6676470.f1.zip › 6676470.f1/[Manuscript] Supplementary Table [S8].docx]

**Supplementary Table S8 Information of target proteins for molecular docking**

| Target name | PDB ID | Resolution(Å) |
| --- | --- | --- |
| CYP3A4 | 5BQG | 1.44 |
| PTAFR | 5ZKQ | 2.90 |
| CNR2 | 5ZTY | 2.80 |
| CNR2 | 6KPC | 3.20 |
| CNR2 | 6KPF | 2.90 |
| FDFT1 | 3ASX | 2.00 |
| FDFT1 | 6PYJ | 1.44 |
| NR3C1 | 4UDD | 1.80 |
| TBXA2R | 6IIU | 2.50 |
| CNR1 | 5TGZ | 2.80 |
| PLA2G1B | 3ELO | 1.55 |
| HSP90AA1 | 4BQG | 1.90 |
| DNMT1 | 4IEJ | 1.45 |
| CYP2C9 | 5TL9 | 1.20 |
| CASP9 | 4RHW | 2.10 |
| ABCB1 | 6C0V | 3.40 |
| ACHE | 4RVK | 1.85 |
| NR3C2 | 4PF3 | 1.10 |
| HMGCR | 2R4F | 1.70 |
| PTPN1 | 4Y14 | 1.90 |
| PTPN2 | 1L8K | 2.56 |
| PTPN7 | 2BIJ | 2.05 |
| PTGDR2 | 6D27 | 2.74 |
