# Supplementary material for: The Exploration of Novel Pharmacophore Characteristics and Multidirectional Elucidation of Structure-Activity Relationship and Mechanism of Sesquiterpene Pyridine Alkaloids from Tripterygium Based on Computational Approaches
Source: Evid Based Complement Alternat Med. 2021 Mar 24;2021:6676470. doi: 10.1155/2021/6676470 (PMC8012133; doi:10.1155/2021/6676470)
Supplement: Supplementary Materials — Supplementary information is available for this paper and listed as follows. Supplementary Table S1: sesquiterpene pyridine alkaloids from Tripterygium classified by structural differences of niacin derivatives. Supplementary Table S2: molecules of pharmacophore model construction and validation for sesquiterpene pyridine alkaloids from Tripterygium. Supplementary Table S3: putative targets of sesquiterpene pyridine alkaloids from Tripterygium. Supplementary Table S4: topological parameters of key targets for sesquiterpene pyridine alkaloids from Tripterygium. Supplementary Table S5: GO enrichment analysis of targets. Supplementary Table S6: KEGG enrichment analysis of targets. Supplementary Table S7: putative diseases of targets for sesquiterpene pyridine alkaloids from Tripterygium. Supplementary Table S8: information of target proteins for molecular docking. Supplementary Table S9: molecular docking results of compound-target pairs ( [file 6676470.f1.zip › 6676470.f1/[Manuscript] Supplementary Table [S9].docx]

**Supplementary Table S9 Molecular docking results of compound-target pairs.**

| Compound | Class | PDB ID of target protein | LibDock Score |
| --- | --- | --- | --- |
| Hypoglaunine E | Type 1 | 5ZKQ | 12.59 |
| Hypoglaunine B  Hypoglaunine B | Type 1 | 5ZKQ | 94.51 |
|  | Type 1 | 6PYJ | 108.79 |
| Hypoglaunine C | Type 1 | 6KPF | 109.49 |
| Hypoglaunine C | Type 1 | 6PYJ | 112.66 |
| Hypoglaunine E | Type 1 | 3ASX | 69.98 |
| Hypoglaunine E | Type 1 | 6PYJ | 126.16 |
| Triptonine B | Type 1 | 6PYJ | 90.36 |
| Wilfordinine B | Type 1 | 6PYJ | 115.53 |
| Wilfordinine C | Type 1 | 6KPF | 77.45 |
| Wilfordinine C | Type 1 | 6PYJ | 132.57 |
| Wilfordinine C | Type 1 | 5TGZ | 104.44 |
| Wilfordinine I | Type 1 | 5ZKQ | 101.27 |
| Wilfordinine I | Type 1 | 3ASX | 114.65 |
| Wilfordinine I | Type 1 | 6PYJ | 107.47 |
| Peritassine A | Type 3 | 6PYJ | 94.01 |
| Wilfornine G | Type 3 | 3ASX | 123.32 |
| Wilfornine G | Type 3 | 6PYJ | 98.85 |
| Cangoronine E-1 | Type 4 | 6PYJ | 91.10 |
| Euojaponine A | Type 4 | 5ZKQ | 79.58 |
| Euojaponine A | Type 4 | 3ASX | 116.35 |
| Euojaponine A | Type 4 | 6PYJ | 118.16 |
| Euojaponine I | Type 4 | 3ASX | 102.32 |
| Euojaponine I | Type 4 | 6PYJ | 107.11 |
| Euojaponine L | Type 4 | 6PYJ | 122.11 |
| Euojaponine M | Type 4 | 6PYJ | 101.16 |
| Euojaponine M | Type 4 | 4BQG | 74.42 |
| Evonine | Type 4 | 3ASX | 81.81 |
| Evonine | Type 4 | 6PYJ | 88.71 |
| Euonymine | Type 4 | 3ASX | 103.28 |
| Euonymine | Type 4 | 6PYJ | 97.41 |
| Forrestine | Type 4 | 5ZKQ | 116.84 |
| Forrestine | Type 4 | 6KPF | 96.08 |
| Forrestine | Type 4 | 6PYJ | 132.77 |
| Hyponine A | Type 4 | 6PYJ | 119.12 |
| Hyponine B | Type 4 | 5ZKQ | 90.61 |
| Hyponine B | Type 4 | 3ASX | 131.75 |
| Hyponine B | Type 4 | 6PYJ | 114.07 |
| Hyponine C | Type 4 | 6PYJ | 102.84 |
| Hyponine D | Type 4 | 5ZKQ | 137.36 |
| Hyponine D | Type 4 | 3ASX | 134.70 |
| Hyponine D | Type 4 | 6PYJ | 127.28 |
| Hyponine E | Type 4 | 3ASX | 114.86 |
| Hyponine E | Type 4 | 6PYJ | 102.73 |
| Hyponine F | Type 4 | 6PYJ | 98.59 |
| Neoeuonymine | Type 4 | 6KPF | 81.34 |
| Neoeuonymine | Type 4 | 3ASX | 97.24 |
| Neoeuonymine | Type 4 | 6PYJ | 115.14 |
| Wilfordinine J | Type 4 | 5ZKQ | 116.11 |
| Wilfordinine J | Type 4 | 3ASX | 92.90 |
| Wilfordinine J | Type 4 | 6PYJ | 115.58 |
| Wilfordinine J | Type 4 | 4BQG | -11.62 |
| Wilfornine F | Type 4 | 3ASX | 67.24 |
| Wilfornine F | Type 4 | 6PYJ | 117.65 |
| Wilfordinine G | Type 5 | 3ASX | 67.67 |
| Wilfordinine G | Type 5 | 6PYJ | 120.87 |
| Wilfordinine H | Type 5 | 6PYJ | 98.91 |
| Alatusinine | Type 6 | 3ASX | 71.23 |
| Alatusinine | Type 6 | 6PYJ | 97.51 |
| Wilfordine | Type 6 | 5ZKQ | 127.89 |
| Wilfordine | Type 6 | 5ZTY | 63.32 |
| Wilfordine | Type 6 | 6KPF | 30.56 |
| Wilfordine | Type 6 | 6PYJ | 119.80 |
| Wilforidine | Type 6 | 3ASX | 123.73 |
| Wilforidine | Type 6 | 6PYJ | 124.86 |
| Wilfornine A | Type 6 | 5ZKQ | 96.40 |
| Wilfornine A | Type 6 | 3ASX | 96.18 |
| Wilfornine A | Type 6 | 6PYJ | 91.78 |
| Wilfornine C | Type 6 | 6KPF | 40.82 |
| Wilfornine C | Type 6 | 6PYJ | 108.12 |
| Wilfornine D | Type 6 | 6KPC | 7.57 |
| Wilfornine D | Type 6 | 3ASX | 52.05 |
| Wilfornine D | Type 6 | 6PYJ | 94.15 |
| Wilfornine E | Type 6 | 6PYJ | 100.81 |
| Wilfortrine | Type 6 | 5ZKQ | -3.58 |
| Wilfortrine | Type 6 | 6KPF | 128.41 |
| Wilfortrine | Type 6 | 3ASX | 108.84 |
| Wilfortrine | Type 6 | 6PYJ | 109.56 |
| Wilfordinine D | Type 7 | 5ZKQ | 53.37 |
| Wilfordinine D | Type 7 | 3ASX | 121.94 |
| Wilfordinine D | Type 7 | 6PYJ | 141.69 |
| Wilfordinine E | Type 7 | 6PYJ | 113.10 |
| Wilfordinine F | Type 7 | 6KPF | 93.44 |
| Wilfordinine F | Type 7 | 3ASX | 83.49 |
| Wilfordinine F | Type 7 | 6PYJ | 109.26 |
| Euojaponine D | Type 8 | 5ZKQ | 59.91 |
| Euojaponine D | Type 8 | 6PYJ | 128.62 |
| Euojaponine D | Type 8 | 4BQG | 74.91 |
| Euojaponine J | Type 8 | 3ASX | 97.53 |
| Euojaponine J | Type 8 | 6PYJ | 101.39 |
| Euojaponine K | Type 8 | 5ZKQ | 98.08 |
| Euojaponine K | Type 8 | 6KPF | 75.92 |
| Euojaponine K | Type 8 | 3ASX | 86.67 |
| Euojaponine K | Type 8 | 6PYJ | 131.35 |
| Wilforgine | Type 8 | 6PYJ | 105.78 |
| Wilforine | Type 8 | 5ZKQ | 92.83 |
| Wilforine | Type 8 | 6PYJ | 134.92 |
| Wilforjine | Type 8 | 6KPF | 77.35 |
| Wilforjine | Type 8 | 3ASX | 102.74 |
| Wilforjine | Type 8 | 6PYJ | 120.19 |
| Wilformine | Type 8 | 5ZKQ | 92.25 |
| Wilformine | Type 8 | 3ASX | 58.56 |
| Wilformine | Type 8 | 6PYJ | 122.54 |
| Wilforzine | Type 8 | 5ZKQ | 94.40 |
| Wilforzine | Type 8 | 3ASX | 122.85 |
| Wilforzine | Type 8 | 6PYJ | 120.14 |
