# Supplementary material for: The Exploration of Novel Pharmacophore Characteristics and Multidirectional Elucidation of Structure-Activity Relationship and Mechanism of Sesquiterpene Pyridine Alkaloids from Tripterygium Based on Computational Approaches
Source: Evid Based Complement Alternat Med. 2021 Mar 24;2021:6676470. doi: 10.1155/2021/6676470 (PMC8012133; doi:10.1155/2021/6676470)
Supplement: Supplementary Materials — Supplementary information is available for this paper and listed as follows. Supplementary Table S1: sesquiterpene pyridine alkaloids from Tripterygium classified by structural differences of niacin derivatives. Supplementary Table S2: molecules of pharmacophore model construction and validation for sesquiterpene pyridine alkaloids from Tripterygium. Supplementary Table S3: putative targets of sesquiterpene pyridine alkaloids from Tripterygium. Supplementary Table S4: topological parameters of key targets for sesquiterpene pyridine alkaloids from Tripterygium. Supplementary Table S5: GO enrichment analysis of targets. Supplementary Table S6: KEGG enrichment analysis of targets. Supplementary Table S7: putative diseases of targets for sesquiterpene pyridine alkaloids from Tripterygium. Supplementary Table S8: information of target proteins for molecular docking. Supplementary Table S9: molecular docking results of compound-target pairs ( [file 6676470.f1.zip › 6676470.f1/Submission-Revised Supplementary Information-20210207.docx]

**Supplementary Information**

**The exploration of novel pharmacophore characteristics and multi-directional elucidation of structure-activity relationship and mechanism of sesquiterpene pyridine alkaloids from Tripterygium based on computational approaches**

**Long *et al*.**

**Supplementary Table S1-S9**

**Supplementary Reference**

**Supplementary Table**

Supplementary Table S1 Sesquiterpene pyridine alkaloids from Tripterygium classified by structural differences of niacin derivatives.

| Num. | Compound | Molecular formula | Class | Reference |
| --- | --- | --- | --- | --- |
| 1 | Hypoglaunine | C_41_H_47_NO_20_ | Type 1 | [1] |
| 2 | Hypoglaunine A | C_41_H_47_NO_20_ | Type 1 | [2] |
| 3 | Hypoglaunine B | C_41_H_47_NO_20_ | Type 1 | [3] |
| 4 | Hypoglaunine C | C_43_H_49_NO_19_ | Type 1 | [4] |
| 5 | Hypoglaunine E | C_39_H_45_NO_19_ | Type 1 | [2] |
| 6 | Triptonine B | C_46_H_50_NO_22_ | Type 1 | [5] |
| 7 | Wilfordinine B | C_38_H_47_NO_19_ | Type 1 | [1] |
| 8 | Wilfordinine C | C_43_H_49_NO_19_ | Type 1 | [1] |
| 9 | Wilfordinine I | C_48_H_51_NO_19_ | Type 1 | [4] |
| 10 | Peritassine A | C_38_H_47_NO_18_ | Type 3 | [5] |
| 11 | Wilfordinine A | C_36_H_45_NO_17_ | Type 3 | [2] |
| 12 | Wilfornine G | C_42_H_48_N_2_O_18_ | Type 3 | [6] |
| 13 | 7-Epi-euojaponine A | C_41_H_47_NO_17_ | Type 4 | [7] |
| 14 | Cangoronine E-1 | C_43_H_49_NO_18_ | Type 4 | [8] |
| 15 | Euojaponine A | C_41_H_47_NO_17_ | Type 4 | [7] |
| 16 | Euojaponine C | C_46_H_49_NO_17_ | Type 4 | [9] |
| 17 | Euojaponine I | C_42_H_48_N_2_O_18_ | Type 4 | Database |
| 18 | Euojaponine L | C_45_H_48_N_2_O_17_ | Type 4 | Database |
| 19 | Euojaponine M | C_40_H_46_N_2_O_17_ | Type 4 | Database |
| 20 | Euonymine | C_38_H_47_NO_18_ | Type 4 | [8] |
| 21 | Evonine | C_36_H_43_NO_17_ | Type 4 | [10] |
| 22 | Forrestine | C_41_H_47_NO_19_ | Type 4 | [11] |
| 23 | Hyponine A | C_41_H_47_NO_19_ | Type 4 | Database |
| 24 | Hyponine B | C_41_H_47_NO_19_ | Type 4 | [12] |
| 25 | Hyponine C | C_43_H_49_NO_18_ | Type 4 | [7] |
| 26 | Hyponine D | C_47_H_50_N_2_O_18_ | Type 4 | [7] |
| 27 | Hyponine E | C_45_H_48_N_2_O_19_ | Type 4 | Database |
| 28 | Hyponine F | C_41_H_47_NO_19_ | Type 4 | Database |
| 29 | Neoeuonymine | C_36_H_45_NO_17_ | Type 4 | [7] |
| 30 | Triptonine A | C_45_H_55_NO_21_ | Type 4 | [12] |
| 31 | Wilfordinine J | C_36_H_45_NO_17_ | Type 4 | [13] |
| 32 | Wilfornine F | C_41_H_47_NO_17_ | Type 4 | [7] |
| 33 | Wilfordinine G | C_36_H_43_NO_16_ | Type 5 | [14] |
| 34 | Wilfordinine H | C_40_H_47_NO_19_ | Type 5 | [14] |
| 35 | Alatusinine | C_38_H_47_NO_19_ | Type 6 | [7] |
| 36 | Wilfordine | C_43_H_49_NO_19_ | Type 6 | [15] |
| 37 | Wilforidine | C_36_H_45_NO_18_ | Type 6 | Database |
| 38 | Wilfornine A | C_45_H_51_NO_20_ | Type 6 | [16] |
| 39 | Wilfornine B | C_43_H_49_NO_19_ | Type 6 | [13] |
| 40 | Wilfornine C | C_50_H_53_NO_20_ | Type 6 | [4] |
| 41 | Wilfornine D | C_43_H_49_NO_21_ | Type 6 | [16] |
| 42 | Wilfornine E | C_36_H_43_NO_18_ | Type 6 | [4] |
| 43 | Wilfortrine | C_41_H_47_NO_20_ | Type 6 | [15] |
| 44 | Wilfordinine D | C_41_H_47_NO_19_ | Type 7 | [14] |
| 45 | Wilfordinine E | C_38_H_47_NO_18_ | Type 7 | [2] |
| 46 | Wilfordinine F | C_43_H_49_NO_18_ | Type 7 | [14] |
| 47 | Euojaponine D | C_41_H_47_NO_17_ | Type 8 | Database |
| 48 | Euojaponine F | C_43_H_49_NO_18_ | Type 8 | [11] |
| 49 | Euojaponine J | C_41_H_47_NO_16_ | Type 8 | Database |
| 50 | Euojaponine K | C_41_H_47_NO_17_ | Type 8 | Database |
| 51 | Wilforgine | C_41_H_47_NO_19_ | Type 8 | [15] |
| 52 | Wilforine | C_43_H_49_NO_18_ | Type 8 | [15] |
| 53 | Wilforjine | C_36_H_45_NO_17_ | Type 8 | [7] |
| 54 | Wilformine | C_38_H_47_NO_18_ | Type 8 | [15] |
| 55 | Wilforzine | C_41_H_47_NO_17_ | Type 8 | [17] |

“Database” represented that related information on sesquiterpene pyridine alkaloids from Tripterygium were obtained from HR-MS-Database of macrocyclic dilactone skeleton alkaloids from Tripterygium established by our research group.

Supplementary Table S2 Molecules of pharmacophore model construction and validation for sesquiterpene pyridine alkaloids from Tripterygium.

| Num. | Name | Active or inactive |
| --- | --- | --- |
| Training set molecule 1 | Wilfordinine I | active |
| Training set molecule 2 | Wilfordinine J | active |
| Training set molecule 3 | Wilfordinine G | active |
| Training set molecule 4 | Wilfornine B | active |
| Training set molecule 5 | Wilfordinine F | active |
| Training set molecule 6 | Euojaponine J | active |
| Decoy set molecule 1 | Hypoglaunine B | active |
| Decoy set molecule 2 | Hypoglaunine C | active |
| Decoy set molecule 3 | Hypoglaunine E | active |
| Decoy set molecule 4 | Triptonine B | active |
| Decoy set molecule 5 | Wilfordinine B | active |
| Decoy set molecule 6 | Wilfordinine C | active |
| Decoy set molecule 7 | Peritassine A | active |
| Decoy set molecule 8 | Wilfornine G | active |
| Decoy set molecule 9 | Cangoronine E-1 | active |
| Decoy set molecule 10 | Euojaponine A | active |
| Decoy set molecule 11 | Euojaponine I | active |
| Decoy set molecule 12 | Euojaponine L | active |
| Decoy set molecule 13 | Euojaponine M | active |
| Decoy set molecule 14 | Euonymine | active |
| Decoy set molecule 15 | Evonine | active |
| Decoy set molecule 16 | Forrestine | active |
| Decoy set molecule 17 | Hyponine A | active |
| Decoy set molecule 18 | Hyponine B | active |
| Decoy set molecule 19 | Hyponine C | active |
| Decoy set molecule 20 | Hyponine E | active |
| Decoy set molecule 21 | Hyponine F | active |
| Decoy set molecule 22 | Neoeuonymine | active |
| Decoy set molecule 23 | Wilfornine F | active |
| Decoy set molecule 24 | Wilfordinine H | active |
| Decoy set molecule 25 | Alatusinine | active |
| Decoy set molecule 26 | Wilfordine | active |
| Decoy set molecule 27 | Wilforidine | active |
| Decoy set molecule 28 | Wilfornine A | active |
| Decoy set molecule 29 | Wilfornine C | active |
| Decoy set molecule 30 | Wilfornine D | active |
| Decoy set molecule 31 | Wilfornine E | active |
| Decoy set molecule 32 | Wilfortrine | active |
| Decoy set molecule 33 | Wilfordinine D | active |
| Decoy set molecule 34 | Wilfordinine E | active |
| Decoy set molecule 35 | Euojaponine D | active |
| Decoy set molecule 36 | Euojaponine K | active |
| Decoy set molecule 37 | Wilforgine | active |
| Decoy set molecule 38 | Wilforine | active |
| Decoy set molecule 39 | Wilforjine | active |
| Decoy set molecule 40 | Wilformine | active |
| Decoy set molecule 41 | Wilforzine | active |
| Decoy set molecule 42 | Hypoglaunine | inactive |
| Decoy set molecule 43 | Hypoglaunine A | inactive |
| Decoy set molecule 44 | Wilfordinine A | inactive |
| Decoy set molecule 45 | 7-Epi-euojaponine A | inactive |
| Decoy set molecule 46 | Euojaponine C | inactive |
| Decoy set molecule 47 | Triptonine A | inactive |
| Decoy set molecule 48 | Euojaponine F | inactive |

Supplementary Table S3 Putative targets of sesquiterpene pyridine alkaloids from Tripterygium.

| Num. | Gene ID | Gene symbol | Gene name |
| --- | --- | --- | --- |
| 1 | 1786 | DNMT1 | DNA methyltransferase 1 |
| 2 | 1268 | CNR1 | cannabinoid receptor 1 |
| 3 | 5770 | PTPN1 | protein tyrosine phosphatase non-receptor type 1 |
| 4 | 5243 | ABCB1 | ATP binding cassette subfamily B member 1 |
| 5 | 5724 | PTAFR | platelet activating factor receptor |
| 6 | 3156 | HMGCR | 3-hydroxy-3-methylglutaryl-CoA reductase |
| 7 | 1269 | CNR2 | cannabinoid receptor 2 |
| 8 | 5771 | PTPN2 | protein tyrosine phosphatase non-receptor type 2 |
| 9 | 4986 | OPRK1 | opioid receptor kappa 1 |
| 10 | 3741 | KCNA5 | potassium voltage-gated channel subfamily A member 5 |
| 11 | 9261 | MAPKAPK2 | MAPK activated protein kinase 2 |
| 12 | 1909 | EDNRA | endothelin receptor type A |
| 13 | 5729 | PTGDR | prostaglandin D2 receptor |
| 14 | 2222 | FDFT1 | farnesyl-diphosphate farnesyltransferase 1 |
| 15 | 43 | ACHE | acetylcholinesterase (Cartwright blood group) |
| 16 | 1576 | CYP3A4 | cytochrome P450 family 3 subfamily A member 4 |
| 17 | 5319 | PLA2G1B | phospholipase A2 group IB |
| 18 | 4306 | NR3C2 | nuclear receptor subfamily 3 group C member 2 |
| 19 | 2908 | NR3C1 | nuclear receptor subfamily 3 group C member 1 |
| 20 | 5142 | PDE4B | phosphodiesterase 4B |
| 21 | 6915 | TBXA2R | thromboxane A2 receptor |
| 22 | 5778 | PTPN7 | protein tyrosine phosphatase non-receptor type 7 |
| 23 | 3320 | HSP90AA1 | heat shock protein 90 alpha family class A member 1 |
| 24 | 1559 | CYP2C9 | cytochrome P450 family 2 subfamily C member 9 |
| 25 | 5241 | PGR | progesterone receptor |
| 26 | 1910 | EDNRB | endothelin receptor type B |
| 27 | 2862 | MLNR | motilin receptor |
| 28 | 440503 | PLIN5 | perilipin 5 |
| 29 | 1991 | ELANE | elastase, neutrophil expressed |
| 30 | 842 | CASP9 | caspase 9 |
| 31 | 994 | CDC25B | cell division cycle 25B |
| 32 | 4889 | NPY5R | neuropeptide Y receptor Y5 |
| 33 | 1586 | CYP17A1 | cytochrome P450 family 17 subfamily A member 1 |
| 34 | 7026 | NR2F2 | nuclear receptor subfamily 2 group F member 2 |
| 35 | 4843 | NOS2 | nitric oxide synthase 2 |
| 36 | 152 | ADRA2C | adrenoceptor alpha 2C |
| 37 | 1394 | CRHR1 | corticotropin releasing hormone receptor 1 |
| 38 | 6524 | SLC5A2 | solute carrier family 5 member 2 |
| 39 | 1020 | CDK5 | cyclin dependent kinase 5 |
| 40 | 6523 | SLC5A1 | solute carrier family 5 member 1 |
| 41 | 596 | BCL2 | BCL2 apoptosis regulator |
| 42 | 4193 | MDM2 | MDM2 proto-oncogene |
| 43 | 1019 | CDK4 | cyclin dependent kinase 4 |
| 44 | 1017 | CDK2 | cyclin dependent kinase 2 |
| 45 | 6095 | RORA | RAR related orphan receptor A |
| 46 | 3738 | KCNA3 | potassium voltage-gated channel subfamily A member 3 |
| 47 | 8767 | RIPK2 | receptor interacting serine/threonine kinase 2 |
| 48 | 207 | AKT1 | AKT serine/threonine kinase 1 |
| 49 | 238 | ALK | ALK receptor tyrosine kinase |
| 50 | 134 | ADORA1 | adenosine A1 receptor |
| 51 | 1520 | CTSS | cathepsin S |
| 52 | 5150 | PDE7A | phosphodiesterase 7A |
| 53 | 3480 | IGF1R | insulin like growth factor 1 receptor |
| 54 | 5294 | PIK3CG | phosphatidylinositol-4,5-bisphosphate 3-kinase catalytic subunit gamma |
| 55 | 4233 | MET | MET proto-oncogene, receptor tyrosine kinase |
| 56 | 135 | ADORA2A | adenosine A2a receptor |
| 57 | 5347 | PLK1 | polo like kinase 1 |
| 58 | 5599 | MAPK8 | mitogen-activated protein kinase 8 |
| 59 | 2932 | GSK3B | glycogen synthase kinase 3 beta |
| 60 | 5139 | PDE3A | phosphodiesterase 3A |
| 61 | 3326 | HSP90AB1 | heat shock protein 90 alpha family class B member 1 |
| 62 | 3551 | IKBKB | inhibitor of nuclear factor kappa B kinase subunit beta |
| 63 | 5579 | PRKCB | protein kinase C beta |
| 64 | 6790 | AURKA | aurora kinase A |
| 65 | 5293 | PIK3CD | phosphatidylinositol-4,5-bisphosphate 3-kinase catalytic subunit delta |
| 66 | 2534 | FYN | FYN proto-oncogene, Src family tyrosine kinase |
| 67 | 5346 | PLIN1 | perilipin 1 |
| 68 | 5979 | RET | ret proto-oncogene |
| 69 | 3172 | HNF4A | hepatocyte nuclear factor 4 alpha |
| 70 | 196 | AHR | aryl hydrocarbon receptor |
| 71 | 5021 | OXTR | oxytocin receptor |
| 72 | 1215 | CMA1 | chymase 1 |
| 73 | 2159 | F10 | coagulation factor X |
| 74 | 200315 | APOBEC3A | apolipoprotein B mRNA editing enzyme catalytic subunit 3A |
| 75 | 1133 | CHRM5 | cholinergic receptor muscarinic 5 |
| 76 | 5582 | PRKCG | protein kinase C gamma |
| 77 | 1132 | CHRM4 | cholinergic receptor muscarinic 4 |
| 78 | 558 | AXL | AXL receptor tyrosine kinase |
| 79 | 5141 | PDE4A | phosphodiesterase 4A |
| 80 | 1812 | DRD1 | dopamine receptor D1 |
| 81 | 2798 | GNRHR | gonadotropin releasing hormone receptor |
| 82 | 774 | CACNA1B | calcium voltage-gated channel subunit alpha1 B |
| 83 | 185 | AGTR1 | angiotensin II receptor type 1 |
| 84 | 5581 | PRKCE | protein kinase C epsilon |
| 85 | 7297 | TYK2 | tyrosine kinase 2 |
| 86 | 3791 | KDR | vascular endothelial growth factor receptor 2 |

Supplementary Table S4 Topological parameters of key targets for sesquiterpene pyridine alkaloids from Tripterygium.

| Gene name | Degree | Betweenness centrality | Closeness centrality | Clustering coefficient |
| --- | --- | --- | --- | --- |
| TBXA2R | 55 | 0.024 | 0.615 | 0 |
| NR3C1 | 55 | 0.024 | 0.615 | 0 |
| FDFT1 | 55 | 0.024 | 0.615 | 0 |
| PTPN2 | 55 | 0.024 | 0.615 | 0 |
| CNR2 | 55 | 0.024 | 0.615 | 0 |
| PTAFR | 55 | 0.024 | 0.615 | 0 |
| PTPN1 | 55 | 0.024 | 0.615 | 0 |
| CYP3A4 | 55 | 0.024 | 0.615 | 0 |
| HSP90AA1 | 54 | 0.022 | 0.610 | 0 |
| PLA2G1B | 54 | 0.022 | 0.610 | 0 |
| CNR1 | 54 | 0.022 | 0.610 | 0 |
| PTGDR2 | 52 | 0.020 | 0.595 | 0 |
| DNMT1 | 51 | 0.018 | 0.595 | 0 |
| CYP2C9 | 50 | 0.017 | 0.586 | 0 |
| CASP9 | 49 | 0.017 | 0.586 | 0 |
| ABCB1 | 47 | 0.017 | 0.572 | 0 |
| ACHE | 49 | 0.016 | 0.576 | 0 |
| NR3C2 | 42 | 0.011 | 0.542 | 0 |
| PTPN7 | 43 | 0.011 | 0.546 | 0 |
| HMGCR | 41 | 0.010 | 0.535 | 0 |

Supplementary Table S5 GO enrichment analysis of targets.

Detailed information was seen in the Text file.

Supplementary Table S6 KEGG enrichment analysis of targets.

Detailed information was seen in the Text file.

Supplementary Table S7 Putative diseases of targets for sesquiterpene pyridine alkaloids from Tripterygium.

| Target gene | Putative disease | Disease number |
| --- | --- | --- |
| CASP9 | Endometrial cancer | D1 |
| AKT1 | Endometrial cancer | D1 |
| GSK3B | Endometrial cancer | D1 |
| PIK3CD | Endometrial cancer | D1 |
| PGR | Breast cancer | D2 |
| CDK4 | Breast cancer | D2 |
| AKT1 | Breast cancer | D2 |
| IGF1R | Breast cancer | D2 |
| GSK3B | Breast cancer | D2 |
| PIK3CD | Breast cancer | D2 |
| NOS2 | Chagas disease (American trypanosomiasis) | D3 |
| AKT1 | Chagas disease (American trypanosomiasis) | D3 |
| MAPK8 | Chagas disease (American trypanosomiasis) | D3 |
| IKBKB | Chagas disease (American trypanosomiasis) | D3 |
| PIK3CD | Chagas disease (American trypanosomiasis) | D3 |
| CASP9 | Alzheimer disease | D4 |
| NOS2 | Alzheimer disease | D4 |
| CDK5 | Alzheimer disease | D4 |
| AKT1 | Alzheimer disease | D4 |
| MAPK8 | Alzheimer disease | D4 |
| GSK3B | Alzheimer disease | D4 |
| IKBKB | Alzheimer disease | D4 |
| PIK3CD | Alzheimer disease | D4 |
| CHRM5 | Alzheimer disease | D4 |
| AKT1 | Non-alcoholic fatty liver disease (NAFLD) | D5 |
| MAPK8 | Non-alcoholic fatty liver disease (NAFLD) | D5 |
| GSK3B | Non-alcoholic fatty liver disease (NAFLD) | D5 |
| IKBKB | Non-alcoholic fatty liver disease (NAFLD) | D5 |
| PIK3CD | Non-alcoholic fatty liver disease (NAFLD) | D5 |
| NOS2 | Amoebiasis | D6 |
| PRKCB | Amoebiasis | D6 |
| PIK3CD | Amoebiasis | D6 |
| PRKCG | Amoebiasis | D6 |
| AKT1 | Acute myeloid leukemia | D7 |
| IKBKB | Acute myeloid leukemia | D7 |
| PIK3CD | Acute myeloid leukemia | D7 |
| AKT1 | Renal cell carcinoma | D8 |
| MET | Renal cell carcinoma | D8 |
| PIK3CD | Renal cell carcinoma | D8 |
| PRKCB | African trypanosomiasis | D9 |
| PRKCG | African trypanosomiasis | D9 |
| MDM2 | Bladder cancer | D10 |
| CDK4 | Bladder cancer | D10 |
| CASP9 | Amyotrophic lateral sclerosis (ALS) | D11 |
| BCL2 | Amyotrophic lateral sclerosis (ALS) | D11 |
| CASP9 | Viral myocarditis | D12 |
| FYN | Viral myocarditis | D12 |
| NOS2 | Pertussis | D13 |
| MAPK8 | Pertussis | D13 |
| NOS2 | Leishmaniasis | D14 |
| PRKCB | Leishmaniasis | D14 |
| HNF4A | Maturity onset diabetes of the young | D15 |
| CASP9 | Parkinson disease | D16 |
| ADORA2A | Parkinson disease | D16 |
| MAPK8 | Parkinson disease | D16 |
| DRD1 | Parkinson disease | D16 |
| FYN | Prion diseases | D17 |
| RET | Thyroid cancer | D18 |
| MET | Malaria | D19 |
| CASP9 | Legionellosis | D20 |
| GSK3B | Basal cell carcinoma | D21 |
| RORA | Inflammatory bowel disease (IBD) | D22 |
| CASP9 | Huntington disease | D23 |
| MAPK8 | Huntington disease | D23 |
| CACNA1B | Huntington disease | D23 |
| ELANE | Systemic lupus erythematosus | D24 |

Supplementary Table S8 Information of target proteins for molecular docking.

| Target name | PDB ID | Resolution(Å) |
| --- | --- | --- |
| CYP3A4 | 5BQG | 1.44 |
| PTAFR | 5ZKQ | 2.90 |
| CNR2 | 5ZTY | 2.80 |
| CNR2 | 6KPC | 3.20 |
| CNR2 | 6KPF | 2.90 |
| FDFT1 | 3ASX | 2.00 |
| FDFT1 | 6PYJ | 1.44 |
| NR3C1 | 4UDD | 1.80 |
| TBXA2R | 6IIU | 2.50 |
| CNR1 | 5TGZ | 2.80 |
| PLA2G1B | 3ELO | 1.55 |
| HSP90AA1 | 4BQG | 1.90 |
| DNMT1 | 4IEJ | 1.45 |
| CYP2C9 | 5TL9 | 1.20 |
| CASP9 | 4RHW | 2.10 |
| ABCB1 | 6C0V | 3.40 |
| ACHE | 4RVK | 1.85 |
| NR3C2 | 4PF3 | 1.10 |
| HMGCR | 2R4F | 1.70 |
| PTPN1 | 4Y14 | 1.90 |
| PTPN2 | 1L8K | 2.56 |
| PTPN7 | 2BIJ | 2.05 |
| PTGDR2 | 6D27 | 2.74 |

Supplementary Table S9 Molecular docking results of compound-target pairs.

| Compound | Class | PDB ID of target protein | LibDock Score |
| --- | --- | --- | --- |
| Hypoglaunine E | Type 1 | 5ZKQ | 12.59 |
| Hypoglaunine B  Hypoglaunine B | Type 1 | 5ZKQ | 94.51 |
|  | Type 1 | 6PYJ | 108.79 |
| Hypoglaunine C | Type 1 | 6KPF | 109.49 |
| Hypoglaunine C | Type 1 | 6PYJ | 112.66 |
| Hypoglaunine E | Type 1 | 3ASX | 69.98 |
| Hypoglaunine E | Type 1 | 6PYJ | 126.16 |
| Triptonine B | Type 1 | 6PYJ | 90.36 |
| Wilfordinine B | Type 1 | 6PYJ | 115.53 |
| Wilfordinine C | Type 1 | 6KPF | 77.45 |
| Wilfordinine C | Type 1 | 6PYJ | 132.57 |
| Wilfordinine C | Type 1 | 5TGZ | 104.44 |
| Wilfordinine I | Type 1 | 5ZKQ | 101.27 |
| Wilfordinine I | Type 1 | 3ASX | 114.65 |
| Wilfordinine I | Type 1 | 6PYJ | 107.47 |
| Peritassine A | Type 3 | 6PYJ | 94.01 |
| Wilfornine G | Type 3 | 3ASX | 123.32 |
| Wilfornine G | Type 3 | 6PYJ | 98.85 |
| Cangoronine E-1 | Type 4 | 6PYJ | 91.10 |
| Euojaponine A | Type 4 | 5ZKQ | 79.58 |
| Euojaponine A | Type 4 | 3ASX | 116.35 |
| Euojaponine A | Type 4 | 6PYJ | 118.16 |
| Euojaponine I | Type 4 | 3ASX | 102.32 |
| Euojaponine I | Type 4 | 6PYJ | 107.11 |
| Euojaponine L | Type 4 | 6PYJ | 122.11 |
| Euojaponine M | Type 4 | 6PYJ | 101.16 |
| Euojaponine M | Type 4 | 4BQG | 74.42 |
| Evonine | Type 4 | 3ASX | 81.81 |
| Evonine | Type 4 | 6PYJ | 88.71 |
| Euonymine | Type 4 | 3ASX | 103.28 |
| Euonymine | Type 4 | 6PYJ | 97.41 |
| Forrestine | Type 4 | 5ZKQ | 116.84 |
| Forrestine | Type 4 | 6KPF | 96.08 |
| Forrestine | Type 4 | 6PYJ | 132.77 |
| Hyponine A | Type 4 | 6PYJ | 119.12 |
| Hyponine B | Type 4 | 5ZKQ | 90.61 |
| Hyponine B | Type 4 | 3ASX | 131.75 |
| Hyponine B | Type 4 | 6PYJ | 114.07 |
| Hyponine C | Type 4 | 6PYJ | 102.84 |
| Hyponine D | Type 4 | 5ZKQ | 137.36 |
| Hyponine D | Type 4 | 3ASX | 134.70 |
| Hyponine D | Type 4 | 6PYJ | 127.28 |
| Hyponine E | Type 4 | 3ASX | 114.86 |
| Hyponine E | Type 4 | 6PYJ | 102.73 |
| Hyponine F | Type 4 | 6PYJ | 98.59 |
| Neoeuonymine | Type 4 | 6KPF | 81.34 |
| Neoeuonymine | Type 4 | 3ASX | 97.24 |
| Neoeuonymine | Type 4 | 6PYJ | 115.14 |
| Wilfordinine J | Type 4 | 5ZKQ | 116.11 |
| Wilfordinine J | Type 4 | 3ASX | 92.90 |
| Wilfordinine J | Type 4 | 6PYJ | 115.58 |
| Wilfordinine J | Type 4 | 4BQG | -11.62 |
| Wilfornine F | Type 4 | 3ASX | 67.24 |
| Wilfornine F | Type 4 | 6PYJ | 117.65 |
| Wilfordinine G | Type 5 | 3ASX | 67.67 |
| Wilfordinine G | Type 5 | 6PYJ | 120.87 |
| Wilfordinine H | Type 5 | 6PYJ | 98.91 |
| Alatusinine | Type 6 | 3ASX | 71.23 |
| Alatusinine | Type 6 | 6PYJ | 97.51 |
| Wilfordine | Type 6 | 5ZKQ | 127.89 |
| Wilfordine | Type 6 | 5ZTY | 63.32 |
| Wilfordine | Type 6 | 6KPF | 30.56 |
| Wilfordine | Type 6 | 6PYJ | 119.80 |
| Wilforidine | Type 6 | 3ASX | 123.73 |
| Wilforidine | Type 6 | 6PYJ | 124.86 |
| Wilfornine A | Type 6 | 5ZKQ | 96.40 |
| Wilfornine A | Type 6 | 3ASX | 96.18 |
| Wilfornine A | Type 6 | 6PYJ | 91.78 |
| Wilfornine C | Type 6 | 6KPF | 40.82 |
| Wilfornine C | Type 6 | 6PYJ | 108.12 |
| Wilfornine D | Type 6 | 6KPC | 7.57 |
| Wilfornine D | Type 6 | 3ASX | 52.05 |
| Wilfornine D | Type 6 | 6PYJ | 94.15 |
| Wilfornine E | Type 6 | 6PYJ | 100.81 |
| Wilfortrine | Type 6 | 5ZKQ | -3.58 |
| Wilfortrine | Type 6 | 6KPF | 128.41 |
| Wilfortrine | Type 6 | 3ASX | 108.84 |
| Wilfortrine | Type 6 | 6PYJ | 109.56 |
| Wilfordinine D | Type 7 | 5ZKQ | 53.37 |
| Wilfordinine D | Type 7 | 3ASX | 121.94 |
| Wilfordinine D | Type 7 | 6PYJ | 141.69 |
| Wilfordinine E | Type 7 | 6PYJ | 113.10 |
| Wilfordinine F | Type 7 | 6KPF | 93.44 |
| Wilfordinine F | Type 7 | 3ASX | 83.49 |
| Wilfordinine F | Type 7 | 6PYJ | 109.26 |
| Euojaponine D | Type 8 | 5ZKQ | 59.91 |
| Euojaponine D | Type 8 | 6PYJ | 128.62 |
| Euojaponine D | Type 8 | 4BQG | 74.91 |
| Euojaponine J | Type 8 | 3ASX | 97.53 |
| Euojaponine J | Type 8 | 6PYJ | 101.39 |
| Euojaponine K | Type 8 | 5ZKQ | 98.08 |
| Euojaponine K | Type 8 | 6KPF | 75.92 |
| Euojaponine K | Type 8 | 3ASX | 86.67 |
| Euojaponine K | Type 8 | 6PYJ | 131.35 |
| Wilforgine | Type 8 | 6PYJ | 105.78 |
| Wilforine | Type 8 | 5ZKQ | 92.83 |
| Wilforine | Type 8 | 6PYJ | 134.92 |
| Wilforjine | Type 8 | 6KPF | 77.35 |
| Wilforjine | Type 8 | 3ASX | 102.74 |
| Wilforjine | Type 8 | 6PYJ | 120.19 |
| Wilformine | Type 8 | 5ZKQ | 92.25 |
| Wilformine | Type 8 | 3ASX | 58.56 |
| Wilformine | Type 8 | 6PYJ | 122.54 |
| Wilforzine | Type 8 | 5ZKQ | 94.40 |
| Wilforzine | Type 8 | 3ASX | 122.85 |
| Wilforzine | Type 8 | 6PYJ | 120.14 |

**Supplementary Reference**

[1] H. Duan, Y. Takaishi, Y. Imakura, et al, “Sesquiterpene alkaloids from *Tripterygium hypoglaucum* and *Tripterygium wilfordii*: a new class of potent anti-HIV agents,” *J Nat Prod*, vol. 63, no. 3, pp. 357-61, 2000.

[2] C. Wang, C. J. Li, J. Ma, et al, “Bioactive sesquiterpene polyol esters from the leaves of *Tripterygium wilfordii*,” *Fitoterapia*, vol. 96, pp. 103-8, 2014.

[3] C. Gao, L. L. Lou, D. Wang, et al, “Chemical constituents from the roots of *Tripterygium wilfordii* and their cytotoxic activity,” *J Asian Nat Prod Res*, vol. 19, no. 7, pp. 725-731, 2017.

[4] H. Duan, Y. Takaishi, H. Momota, et al, “Immunosuppressive sesquiterpene alkaloids from *Tripterygium wilfordii*,” *J Nat Prod*, vol. 64, no. 5, pp. 582-7, 2001.

[5] X. Du, X. He, Y. H. Huang, et al, “Simultaneous determination of seven effective components of Tripterygium glycosides in human biological matrices by ultra-performance liquid chromatography-triple quadrupole mass spectrometry,” *J Chromatogr B Analyt Technol Biomed Life Sci*, vol. 1113, pp. 1-13, 2019.

[6] Y. G. Luo, M. Zhou, Q. Ye, et al, “Dihydroagarofuran derivatives from the dried roots of *Tripterygium wilfordii*,” *J Nat Prod*, vol. 75, no. 1, pp. 98-102, 2012.

[7] Y. G. Luo, X. Pu, G. Y. Luo, et al, “Nitrogen-containing dihydro-β-agarofuran derivatives from *Tripterygium wilfordii*,” *J Nat Prod*, vol. 77, no. 7, pp. 1650-7, 2014.

[8] T. Cai, Y. G. Luo, M. Zhou, et al, “Untargeted analysis of sesquiterpene pyridine alkaloids from the dried roots of *Tripterygium wilfordii* using high-performance liquid chromatography/electrospray ionization tandem mass spectrometry,” *Rapid Commun Mass Spectrom*, vol. 29, no. 10, pp. 965-72, 2015.

[9] J. H. Ryu, S. Y. Ryu, Y. N. Han, B. H. Han, “Absolute configuration of beta-agarofuran nucleus of euojaponine C by CD exciton chirality method,” *Arch Pharm Res*, vol. 20, no. 1, pp. 76-9, 1997.

[10] M. Q. Cai, S. W. He, Y. J. Shi, et al, “Rapid and sensitive analysis of euonine and wilforidine in human plasma by high-performance liquid chromatography-atmospheric-pressure chemical ionization-mass spectrometry,” *J Anal Toxicol*, vol. 37, no. 7, pp. 395-400, 2013.

[11] L. M. Lião, P. C. Vieira, E. Rodrigues-Filho, J. B. Fernandes, M. F. da Silva, “Sesquiterpene pyridine alkaloids from *Peritassa campestris*,” *Phytochemistry*, vol. 58, no. 8, pp. 1205-7, 2001.

[12] J. Q. Liu, “A method of processing *Tripterygium wilfordii*, CN104306426A, 2015-01-28.

[13] H. Duan, Y. Takaishi, H. Momota, et al, “Immunosuppressive sesquiterpene alkaloids from *Tripterygium wilfordii*,” *J Nat Prod*, vol. 64, no. 5, pp. 582-7, 2001.

[14] J. Z. Xu, J. Lu, F. Sun, et al, “Terpenoids from *Tripterygium wilfordii*,” *Phytochemistry*, vol. 72, no. 11, pp. 1482-7, 2011.

[15] H. T. Guo, Z. Y. Wang, L. Y. Xu, et al, “Separation and simultaneous determination of seven bioactive components in *Tripterygium wilfordii* Hook. F. and Tripterygium preparations by micellar electrokinetic capillary chromatography,” *Electrophoresis*, vol. 40, no. 4, pp. 547-554, 2019.

[16] C. Gao, X. X. Huang, M. Bai, et al, “Anti-inflammatory sesquiterpene pyridine alkaloids from *Tripterygium wilfordii*,” *Fitoterapia*, vol. 105, pp. 49-54, 2015.

[17] Y. L. Chen, X. Liu, X. Y. Qu, et al, “Studies on difference of chemical compositions in plant species of Tripterygium genus,” *Zhongguo Zhong Yao Za Zhi*, vol. 42, no.2, pp. 319-325, 2017.
